# Caffeine and Cationic Copolymers with Antimicrobial Properties

**DOI:** 10.1021/acsbiomedchemau.2c00077

**Published:** 2023-02-13

**Authors:** Pedro Salas-Ambrosio, Shelby Vexler, Rajalakshmi P S, Irene A. Chen, Heather D. Maynard

**Affiliations:** † Department of Chemistry and Biochemistry and California Nano Systems Institute, University of California, Los Angeles, 607 Charles E. Young Drive East, Los Angeles, California 90095, United States; ‡ Department of Chemical and Biomolecular Engineering, University of California, Los Angeles, 508 Portola Plaza, Los Angeles, California 90095, United States

**Keywords:** Cationic polymethacrylates, antimicrobial polymers, primary ammonium, quaternary ammonium, caffeine polymers

## Abstract

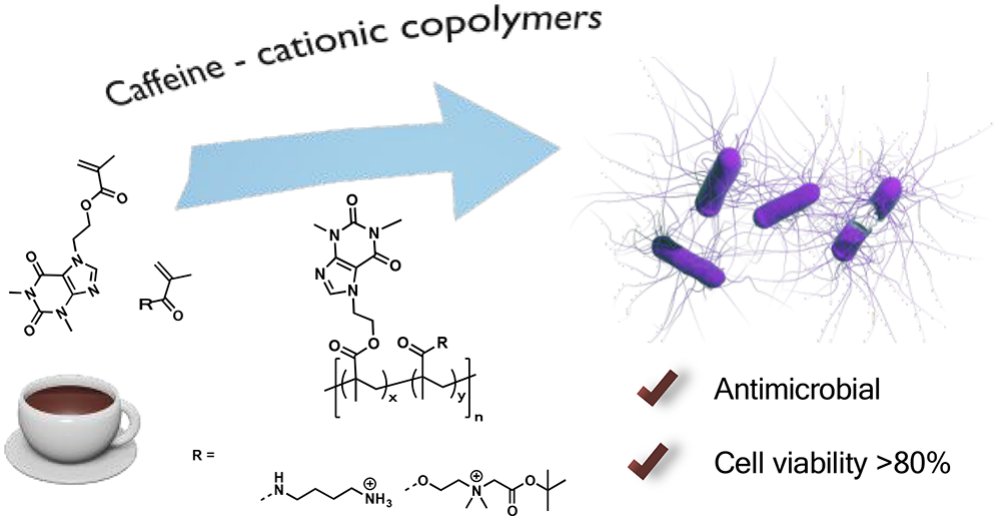

One of the primary global health concerns is the increase in antimicrobial resistance. Polymer chemistry enables the preparation of macromolecules with hydrophobic and cationic side chains that kill bacteria by destabilizing their membranes. In the current study, macromolecules are prepared by radical copolymerization of caffeine methacrylate as the hydrophobic monomer and cationic- or zwitterionic-methacrylate monomers. The synthesized copolymers bearing *tert*-butyl-protected carboxybetaine as cationic side chains showed antibacterial activity toward Gram-positive bacteria (*S. aureus*) and Gram-negative bacteria (*E. coli*). By tuning the hydrophobic content, we prepared copolymers with optimal antibacterial activity against *S. aureus*, including methicillin-resistant clinical isolates. Moreover, the caffeine–cationic copolymers presented good biocompatibility in a mouse embryonic fibroblast cell line, NIH 3T3, and hemocompatibility with erythrocytes even at high hydrophobic monomer content (30–50%). Therefore, incorporating caffeine and introducing *tert*-butyl-protected carboxybetaine as a quaternary cation in polymers could be a novel strategy to combat bacteria.

## Introduction

Antimicrobial resistance (AMR) poses a rising concern to human health as bacteria continually evolve novel mechanisms to defeat antibiotics.^1,2^ According to the World Health Organization in 2020, this global issue is exacerbated by the misuse or overuse of existing antibiotics, along with a lack of investment in technology development to combat AMR.^3^ One promising area of research is the development of antimicrobial peptides (AMPs), which have been widely reported to fight drug-resistant microbes.^4−7^ AMPs are small molecules constituted of amino acids either found naturally or prepared synthetically.^8^ Their characteristic antimicrobial activity is determined by the ratio of cationic to hydrophobic amino acids which promotes bacterial membrane disruption.^9−12^ However, the practical use of AMPs is restricted by scalability, high cost of production, and the multistep manufacturing process.^9,10^ One of the solutions is to use polymer chemistry to synthesize macromolecules that mimic the structural features of AMPs, endowing them with the same antimicrobial properties.^11−13^ This class of polymers, also known as polymer biocides,^14^ can be prepared from a variety of different polymerization techniques depending on the nature of the monomers. Among the different polymeric backbones with antimicrobial properties, it is possible to find polyacrylates,^15^ polynorbornenes,^16^ polypeptides,^17^ their analogues polypeptoids,^18,19^ or synthetic polybetapeptides.^20^ Different parameters can be tuned during the preparation of antimicrobial polymers, such as architecture, molecular weight, monomer arrangement, nature of the cation, and hydrophobic group.^11,21^ Among the polymer biocides, modifications include zwitterionic groups as side chains, and satellite-active groups.^22,23^ Particularly, the cation structure and hydrophobic side chain play an important role in antimicrobial peptides.^24−26^ Within the different polymer backbones, poly(methacrylates) can be tuned at the side chain to mimic amino acid cationic groups.^27,28^ These polymers are nondegradable in body conditions,^29^ in comparison to other polymers; thus biodistribution and excretion studies are important for translation.^30^ A key parameter for presenting antibacterial activity is the nature of the cation, and for poly(methacrylates), the primary cationic group has shown to be more effective than permanent quaternary cationic groups, even if both can interact with the negatively charged species over bacterial membranes.^31,32^ Increased length of the hydrophobic side chain allows for greater hydrophobic interactions with the lipid components of the membranes but comes at the cost of higher cytotoxicity.^33^ Therefore, evaluating different ratios of cationic and hydrophobic content is crucial to select molecules with optimal antimicrobial activity and minimal cytotoxicity.

**Scheme 1:**
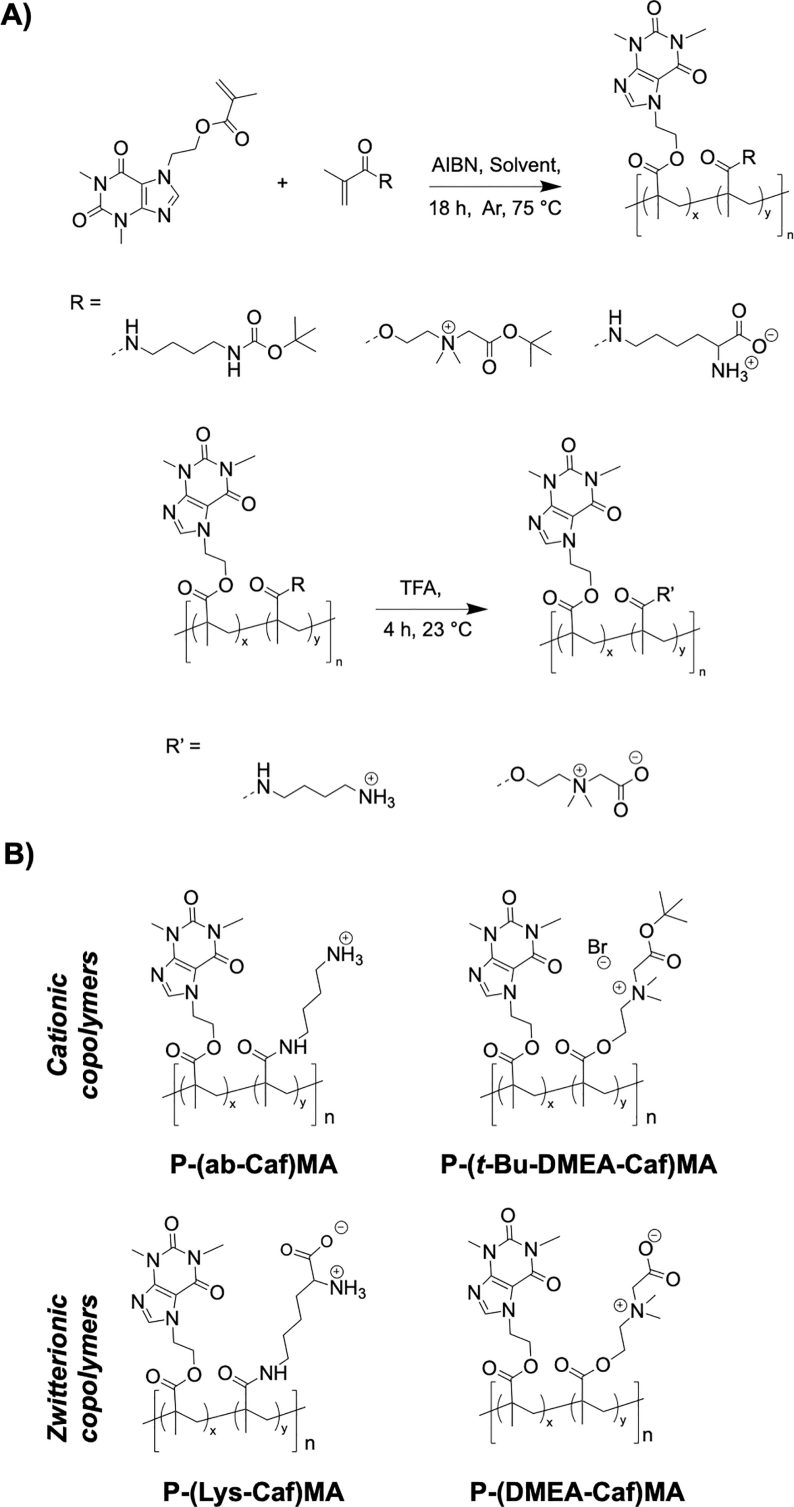
General Strategy to Prepare Methacrylate Copolymers with Caffeine and Cationic or Zwitterionic Side Chains: (A) Synthetic Route of Preparation and (B) Copolymer Structures

Moreover, to the best of our knowledge, heterocyclic side chains, such as purine derivatives, have not been reported as the hydrophobic side chain of antimicrobial polymers, whereas polymers having heterocyclic cationic side chains are already known.^34−37^ Potential purine derivatives include caffeine, which could provide antibacterial properties by itself^38,39^ or improve the biological activity of other drugs.^40,41^ In the current study described here, we prepared a series of methacrylate copolymers bearing cationic or zwitterionic groups and ethyl caffeine by free-radical polymerization and tested their potential as antimicrobial agents (Scheme 1).

## Results and Discussion

### Polymer Preparation

To investigate the antibacterial properties of caffeine copolymers with hydrophilic charged groups, we prepared libraries of random copolymers through free-radical polymerization. Caffeine polymer derivatives are known to be accessible by free radical polymerization through methacrylate functionalization at the 8-position^42^ or 7-position^43^ of the caffeine heterocycle or by polymerization of lipoylated caffeine.^44^ In the current study, we used etofylline, a derivative of caffeine bearing an ethyl alcohol side chain at the 7-position and functionalized with a methacrylate (Caf-MA) by nucleophilic substitution of methacryloyl chloride in a 56% yield (Figures S1 and S2). Free-radical homopolymerization of Caf-MA (30 equiv) was performed using azobis(isobutyronitrile) (AIBN, 1 equiv) in dimethyl sulfoxide (DMSO) under argon at 75 °C. After 100 min, the polymer poly(etofylline methacrylate) (P(Caf-MA)) was isolated by precipitation in ethyl acetate and characterized by ^1^H NMR and size exclusion chromatography (SEC) in trifluoroethanol (TFE), showing a molecular weight by number *M*_*n*_ = 25.4 kg/mol and molecular weight dispersity *Đ* = 2.77 (Figures S3 and S4). The insolubility in phosphate buffer saline (PBS) at 1 mg/mL, and the calculated logP of monomer and small oligomers, demonstrated its hydrophobic character (Figure S5).

**Figure 1:**
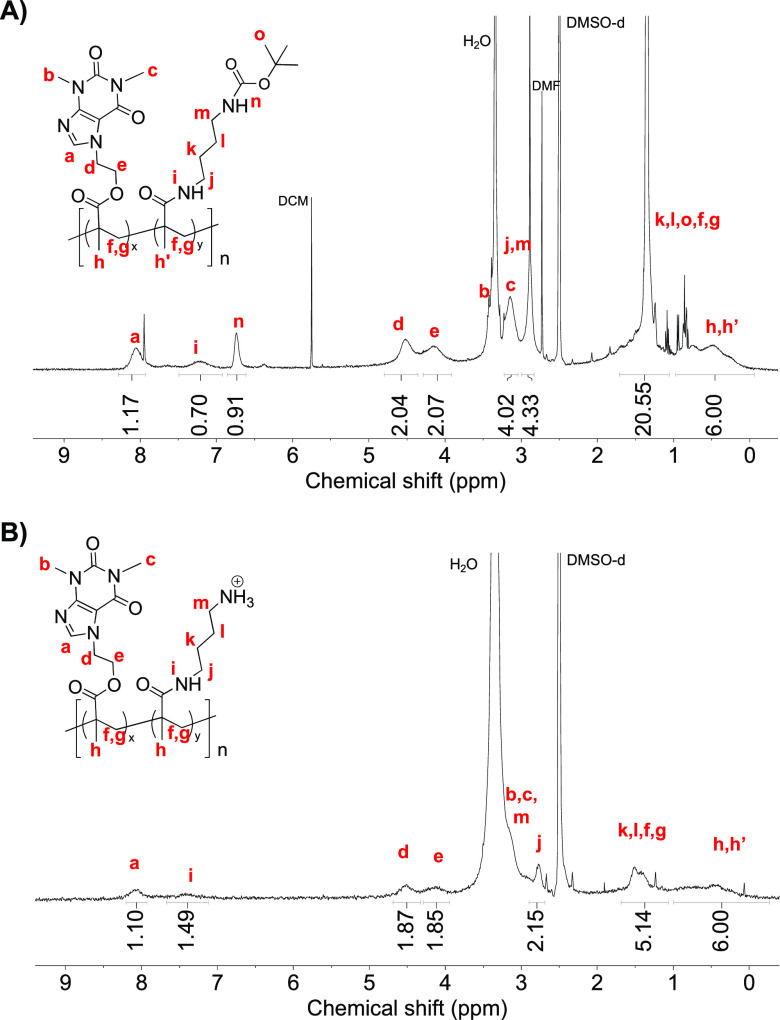
^1^H NMR of a representative copolymer bearing caffeine and cationic side chains (A) before (P-(Boc-ab-Caf 50%)MA) and (B) upon acidic deprotection (P-(ab-Caf 50%)MA).

It is known that the cationic-hydrophobic ratio as well as the nature of these side chains are important parameters during the preparation of polymers to display antimicrobial activity.^11,21^ Therefore, we prepared copolymer libraries of P(Caf-MA) through a similar methodology, varying the caffeine content from 50% to 0% and using monomers with hydrophilic side chains. To obtain copolymers with cationic side chains and caffeine, the monomer *tert*-butyl (4-methacrylamidobutyl) carbamate (Boc-ab-MA) was prepared by nucleophilic substitution of methacryloyl chloride and *tert*-butyl(4-aminobutyl) carbamate in a 65% yield (Figure S6 and S7). Then, free-radical polymerization was performed in dimethylformamide (DMF) under inert atmosphere conditions at 75 °C using AIBN and varying the content of CafMA from 50 to 0%. The library of P-(Boc-ab-Caf)MA was successfully prepared in a 67–73% yield and the synthesized polymers were characterized by ^1^H NMR and SEC in DMF (Figures 1 and S8–S11).

**Figure 2:**
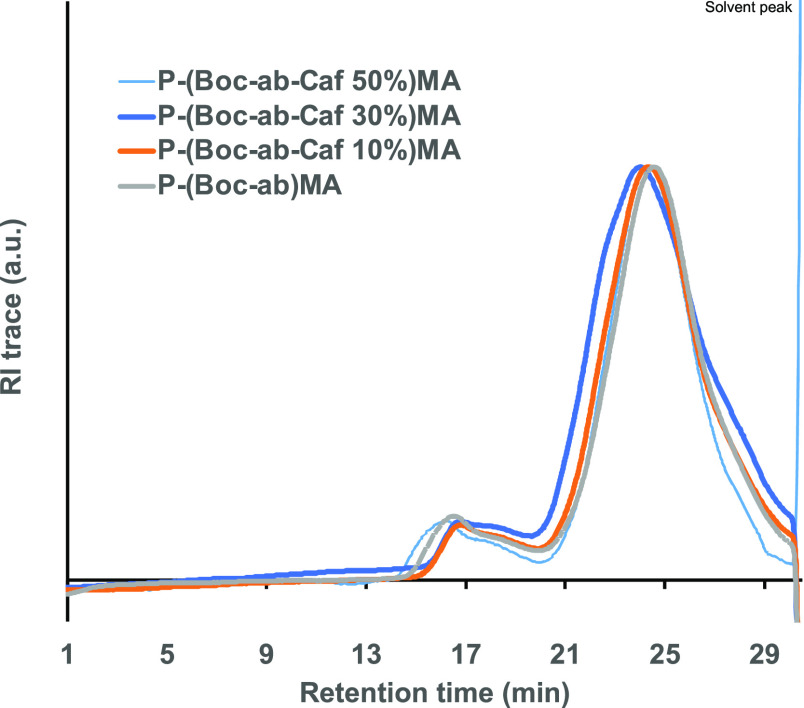
Representative chromatograms from refractive index (RI) trace of copolymer bearing caffeine and cationic side chains from SEC in DMF (au = arbitrary units).

The caffeine content was determined using the signal corresponding to the proton in the heterocyclic moiety (signal *a*, 8.05 ppm) and the protons of the CH_3_- group (signal *h,h’*, 0.49 ppm) as illustrated for the copolymer bearing *N*-Boc aminobutyl and caffeine side chains having a caffeine content of 50% (P-(Boc-ab-Caf 50%)MA, Figure 1). The caffeine content of the copolymers corroborated the theoretical values, according to the ^1^H NMR analyses and had *M*_*n*_ = 21.8–27.7 kg/mol with a polymer dispersity of *Đ* = 1.50–1.75 (Table 1, Figures 1 and 2). A shoulder peak was observed at a lower retention time in the SEC that we attributed to aggregation under the analytic conditions. This interpretation was further confirmed by the light scattering signal from SEC in DMF (Figure S12).

**Table 1:** Characterization of the Copolymers Bearing Caffeine and Cationic or Zwitterionic Side Chains by ^1^H-NMR, SEC, and Yields

polymer	caffeine content theoretical (%)	caffeine content from ^1^H NMR (%)	*M*_*n*_ (kg/mol)	*Đ*	yield (%)
P(Caf)MA	100	100	25.4^a^	2.77^a^	77
P-(ab-Caf 50%)MA	55	55	27.7^b^	1.75^b^	69
P-(ab-Caf 30%)MA	30	34	21.8^b^	1.74^b^	70
P-(ab-Caf 10%)MA	10	10	25.8^b^	1.50^b^	81
P-(ab)MA	0	0	23.2^b^	1.52^b^	73
P-(*t*-Bu -DMEA-Caf 30%)MA^c^	30	26			80
P-(*t-*Bu -DMEA-Caf 10%)MA	10	8	21.1^d^	2.03^d^	83
P-(*t-*Bu-DMEA)MA	0	0	32^d^	2.06^d^	92
P-(DMEA-Caf 30%)MA	30	37	16^d^	2.24^d^	91
P-(DMEA-Caf 10%)MA	10	11	26.7^d^	2.53^d^	86
P-(DMEA)MA	0	0	22.7^d^	3.24^d^	91
P-(Lys-Caf 50%)MA^c^	55	59			4
P-(Lys-Caf 30%)MA	30	38	18^d^	2.24^d^	39
P-(Lys-Caf 10%)MA	10	10	17.8^d^	2.25^d^	38
P-(Lys)MA	0	0	25.2^d^	2.28^d^	17

a Determined from SEC in trifluoroethanol.

b Determined from the Boc-protected polymer through SEC in DMF.

c Polymers were not possible to analyze due to poor solubility under the conditions of analysis.

d Determined from SEC in water.

Further, *N*-Boc deprotection using trifluoroacetic acid (TFA) permitted isolation of the P-(ab-Caf)MA copolymers bearing caffeine and primary ammonium side chains in 69–81% yields. The characterization by SEC was not performed since the polymers were not soluble in the aqueous eluent system. The caffeine content was determined using the signal corresponding to the proton in the heterocyclic moiety (signal *a*, 8.05 ppm) and the protons of the CH_3_- group (signal *h,h’*, 0.49 ppm) as illustrated for the copolymer bearing aminobutyl and caffeine side chains with a 50% of caffeine content (P-(ab-Caf 50%)MA, Figures S13–S16). The caffeine content was consistent with the values before deprotection showing that the deprotection did not cause side chain cleavage.

With the purpose of comparing the activity of the cationic side chain, we prepared copolymers bearing permanent cationic groups, specifically quaternary ammonium, and caffeine side chains P-(*t*-Bu-DMEA-Caf)MA. First, the monomer 2-(*tert*-butoxy)-*N*-(2-(methacryloyloxy)ethyl)-*N,N*-dimethyl-2-oxoethan-1-aminium (*t*-Bu-DMEA-MA) was prepared by quaternization of 2-(dimethylamino) ethyl methacrylate with *tert*-butyl 2-bromoacetate in acetonitrile at 50 °C for 16 h in an 83% yield (Figure S17). Then, free radical copolymerization with AIBN in DMSO was performed using *t*-Bu-DMEA-MA and CafMA while varying the caffeine content from 30 to 0% under inert atmosphere conditions at 75 °C. The copolymers were isolated in 80–92% yield. The copolymers with higher content (>50%) of caffeine were not prepared because the reaction mixture became heterogeneous, and no conversion was observed by ^1^H NMR. ^1^H NMR and SEC were used to characterize the copolymers in water (Figures S18–S21). The caffeine content was determined in a similar way as P-(Boc-ab-Caf)MA and correlated with the theoretical values (Table 1), having *M*_*n*_ = 32.0–21.1 kg/mol and *Đ* = 2.03–2.06. Further, *tert*-butyl ester (*t*-Bu) deprotection in TFA at 23 °C for 4 h allowed us to isolate the copolymers bearing zwitterionic and caffeine side chains P-(DMEA-Caf)MA in 86–91% yields. We characterized the copolymers by ^1^H NMR and SEC in water and the caffeine content was determined in a similar way as for P-(ab-Caf)MA; the values agreed with the values before deprotection, having *M*_*n*_ = 16.0–26.7 kg/mol and *Đ* = 2.24–3.24 (Table 1, Figures S22–S25).

In addition, we prepared another copolymer library bearing zwitterionic groups from amino acid derivatives and caffeine side chains, specifically P-(Lys-Caf)MA. The monomer was prepared by nucleophilic substitution of methacryloyl chloride with *N*-Cbz-Lysine and further *N*-Boc deprotection using HBr and TFA at 23 °C for 4 h, without isolation of the intermediate, which allowed us to obtain lysine methacrylate (LysMA) in a 30% yield (Figure S26). We attributed the low yield to autopolymerization encountered during the preparation of the intermediate. Then we prepared copolymers by free radical polymerization using AIBN in DMSO-water (1:1 v/v) under inert atmosphere conditions at 75 °C for 16 h. We synthesized the library of copolymers in 4–39% yields and characterized the copolymers by ^1^H NMR and SEC in water (Figures S27–S31). We determined the caffeine content similarly to P-(ab-Caf)MA, and the caffeine content agreed with the values before deprotection having *M*_*n*_ = 18.0–25.2 kg/mol with a polymer dispersity of *Đ* = 2.24–2.28 (Table 1).

### Antimicrobial Evaluation

Once the libraries of copolymers were prepared, their activities were tested against the Gram-positive bacteria *S. aureus* (Newman strain) and the Gram-negative bacteria *E. coli* (ATCC 27065). A microdilution method in a 96-well plate was used to determine the minimum inhibitory concentration (MIC), the minimum concentration where no bacterial growth was detectable.^45^ Copolymers bearing the primary cationic group and caffeine (P-(ab-Caf)MA) did not present antimicrobial activity at 50% to 0% of caffeine content (MIC > 500 μg/mL, Tables 2 and S1) against *S. aureus*. In contrast, an increase in anti-infective activity was observed toward *E. coli* when there was a decrease in the caffeine content. The copolymers with 50% caffeine content showed a MIC = 125 μg/mL, whereas copolymers with less caffeine content were more active with MIC = 26–18 μg/mL. The most active compounds were the ones that contained 10% caffeine (P-(ab-Caf 10%)MA) or the cationic homopolymer (P-(ab)MA) having MIC = 19 ± 7 and 18 ± 7 μg/mL, respectively. These results demonstrated that the cationic copolymers were capable of inhibiting the growth of exclusively Gram-negative bacteria, which is related to the simplicity of the membranes as compared to Gram-positive microorganisms.

**Table 2:** Antimicrobial Evaluation of the Caffeine Copolymers against *S. aureus* and *E. coli* through the Microdilution Method by Varying the Caffeine Content (Caf%) and the Hydrophilic Side Chain (*n* = 6)

polymer	*S. aureus* MIC (μg/mL)	*E. coli* MIC (μg/mL)
P-(ab-Caf 50%)MA	>500	125
P-(ab-Caf 30%)MA	>500	26 ± 2
P-(ab-Caf 10%)MA	>500	19 ± 7
P-(ab)MA	>500	18 ± 7
P-(*t-*Bu-DMEA-Caf 30%)MA	>500	125
P-(*t*-Bu-DMEA-Caf 10%)MA	60 ± 39	39 ± 11
P-(*t*-Bu-DMEA)MA^a^	15 ± 7	41 ± 13
Ampicillin	1	15.6
Kanamycin	2	2

The anti-infective activity of the methacrylate copolymers series bearing caffeine and quaternary ammonium side chains (P-(*t*-Bu-Caf)MA) was evaluated using the same methodology (Table 2). The anti-infective activity against *S. aureus* increased as the caffeine content decreased, with MIC = 15 ± 7 μg/mL for the homopolymer (P-(*t*-Bu-DMEA)MA). However, the copolymer with 10% of caffeine (P-(*t*-Bu-DMEA-Caf10%)MA) also demonstrated activity against *S. aureus* having a MIC = 60 ± 39 μg/mL. These values were superior to the activity of P(ab-Caf30%)MA because of the permanent quaternary ammonium group.^11^ A similar trend was noted against *E. coli*; copolymers displayed the best activity with 10% or 0% caffeine, having MIC = 39 ± 11 or 41 ± 13 μg/mL, respectively. The copolymers at 50% caffeine content presented MIC = 125 μg/mL, which was comparable to that of P-(ab-Caf50%)MA. The copolymer series bearing zwitterionic groups, P-(DMEA-Caf)MA and P-(Lys-Caf)MA, did not display anti-infective activity against *S. aureus* or *E. coli*, confirming the necessity of a cationic side chain to inhibit the bacteria growth (Table S1).^45^ To summarize, the copolymers P-(*t*-Bu-DMEA-Caf)MA possess a broad spectrum of action, while primary cationic copolymers were only active against *E. coli*.

It has been shown for other polymethacrylates that the extent of antimicrobial activity depends on molecular weight and that the optimal degree of polymerization is 30.^11^ Therefore, we prepared a random copolymer library having a DP of 30 by reversible addition–fragmentation chain-transfer (RAFT) polymerization. 4-Cyano-4-(phenyl carbonothioyllthio) pentanoic acid (CTA) was employed as the RAFT agent and AIBN as the initiator in DMSO at 75 °C for 16 h. The copolymer library containing caffeine from 30 to 0% was isolated in a 70–76% yield. Upon isolation, the copolymers were characterized by ^1^H NMR and SEC in water (Figures S32–S35 ). The polymerization degree was determined by ^1^H NMR in DMSO using the signals of the aromatic protons in the CTA (signal *o*, 7.46–7.86 ppm) and the signals of the CH_3_ group of the polymer backbone (signal *c*, 0.78 ppm). Then, the caffeine content was determined by correlating the signal of the proton of the caffeine (signal *f*, 8.3 ppm) and the polymerization degree. The polymerization degree and caffeine content correlated with the theoretical value for all the copolymers and displayed *M*_*n*_ = 4.7–6.4 kg/mol and narrow polymer dispersity *Đ* = 1.03–1.16 (Table S2). Further *N*-Boc deprotection in TFA allowed the isolation of P(DMEA-Caf)MA zwitterionic copolymers in a 72–74% yield. The copolymers were characterized by ^1^H NMR and SEC, and the caffeine content and polymerization degree were determined similarly to before deprotection. We observed that the polymers correlated with the DP and the caffeine content before deprotection, and they presented *M*_*n*_ = 4.2–5.1 kg/mol and narrow polymer dispersity *Đ* = 1.16–1.18 (Table S2, Figures S36–S40). These results demonstrated that caffeine methacrylate copolymers bearing cationic or zwitterionic side chains are also accessible through RAFT polymerization with control of the molecular weight and polymer dispersity. Then, the antimicrobial activity was evaluated against *S. aureus* and *E. coli* using the microdilution methodology as previously discussed. Surprisingly, none of the copolymers were active against the bacteria tested (MIC > 500 μg/mL), suggesting that activity is only present at the higher molecular weight (DP > 30). A full molecular weight dependence on antimicrobial activity will need to be studied in the future. The polymerization kinetics of certain methacrylates and methacrylamides have been reported to be similar,^46^ and thus, it is possible that the polymers reported here are random copolymers; this will also need to be investigated in future studies, particularly because sequence could be a major factor in activity.

We selected the two copolymers with the best activity against the Newman strain for further testing against *S. aureus*. As copolymers can be effective against drug-resistant pathogens,^38,39^ we obtained two clinical isolates of methicillin-resistant *S. aureus* (MRSA)^41,42^ and applied the same microdilution method to determine the MIC: 194 μg/mL (P-(*t*-Bu-DMEA-Caf 10%)MA) and 170 μg/mL (P-(*t*-Bu-DMEA)MA, Table S1). Notably, the MICs differed greatly between the two isolates, resulting in a high standard deviation (101 and 105, respectively), yet both copolymers still proved more effective than ampicillin (MIC = 625 μg/mL). While further testing is necessary to assess the clinical relevancy, our initial findings establish the copolymers with caffeine side chains and a bulky hydrophobic side chain on the quaternary ammonium groups as interesting antimicrobial materials.

### Cytocompatibility and Hemocompatibility

**Figure 3:**
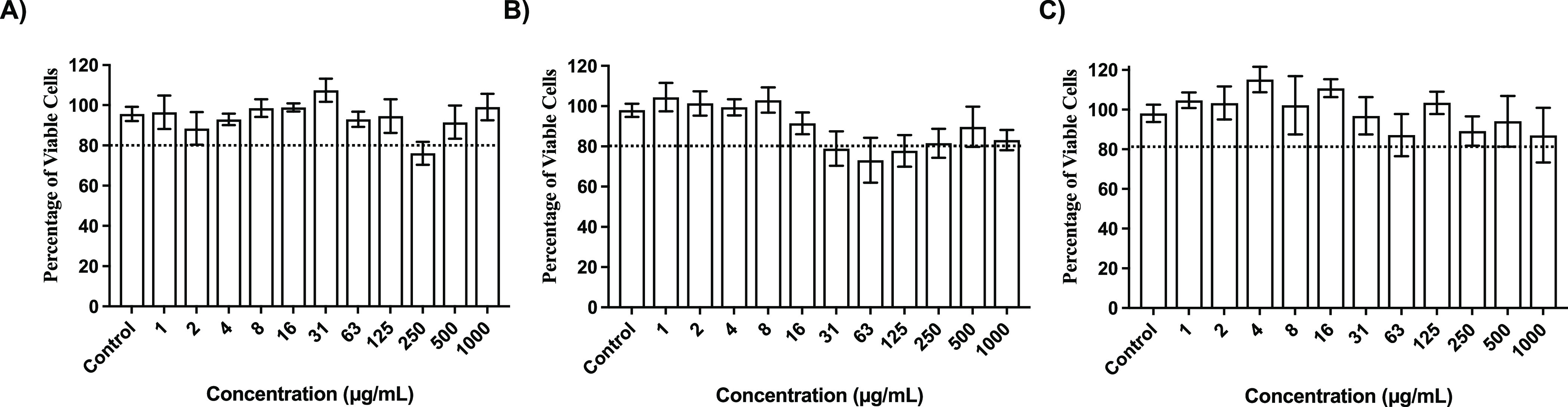
Cytotoxicity of active compounds with broad antibacterial activity: (a) P-(*t*-Bu-DMEA-Caf30%)MA, (b) P-(*t*-Bu-DMEA-Caf10%)MA, and (c) P-(*t*-Bu-DMEA)MA. Data are represented as mean ± SEM (*n* = 5).

To understand the safety profile of the copolymers, we evaluated the cytotoxicity of the active copolymer series P-(ab-Caf)MA and P-(*t*-Bu-DMEA-Caf)MA at different caffeine content in mice fibroblasts (NIH 3T3) at 37 °C for 24 h by using the colorimetric MTT assay (Figures 3 and S41). The copolymers P-(ab-Caf)MA showed enhanced biocompatibility by increasing the caffeine content on the copolymers as follows. With 0 or 10% caffeine (P-(ab-Caf)MA and P-(ab-Caf 10%)MA), cell viability dropped below 80% at polymer concentrations above 31 μg/mL, which is close to the MIC values and not safe for therapeutic purposes (Figure S41). However, at 30% and 50% caffeine content (P-(ab-Caf 30%)MA and P-(ab-Caf 50%)MA), cells tolerated greater polymer concentrations, with cell viability dropping below 80% at 250 or 500 μg/mL, respectively. These results suggested a dependence effect between the caffeine content and the cell viability. Moreover, the results correlated with the antimicrobial activity against *E. coli* where a higher caffeine content led to a reduced inhibitory concentration (i.e., greater potency) as compared to the copolymer without caffeine as a side-chain. Interestingly, when we evaluated the copolymers P-(*t*-Bu-DMEA-Caf)MA, the cell viability was above 80%, even at the highest concentration tested (1000 μg/mL), including for the deprotected zwitterionic form (Figures 3 and S42, respectively). These results confirmed an increase in the biocompatibility promoted by the caffeine side chain for this series of polymers. This suggests an interesting approach that can enhance the biocompatibility while preserving the antimicrobial properties of quaternary ammonium copolymers that were often reported to be toxic in the literature even if in the literature previously were often toxic at a hydrophobic content >30%.^32,47,48^ This particular behavior can be attributed to the purine nature of the hydrophobic side chain as it is reported that it can decrease cytotoxicity during the preparation of other drugs.^40,41^ Moreover, the use of *tert*-butyl protected carboxybetaine as part of the structure of the copolymer can be used to explore other hydrophobic side chains, as the homopolymer presented cell viability > 80%.

**Figure 4:**
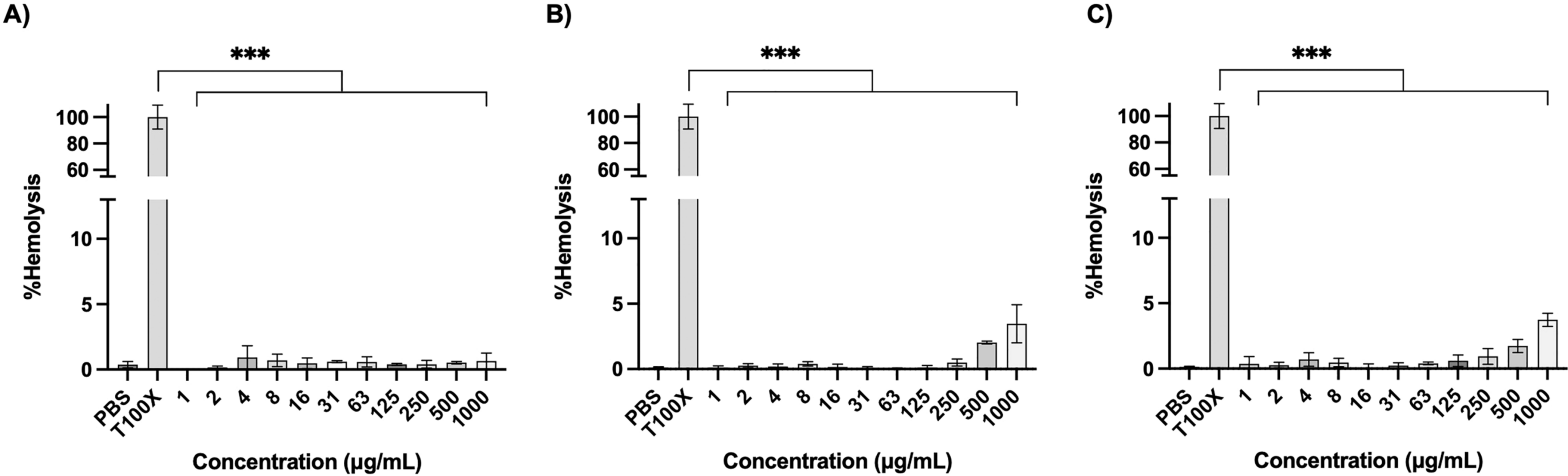
Representative hemolytic effect of anti-infective polymers determined at 450 nm in sheep erythrocytes at 37 °C for 1 h using PBS and Triton X-100 (T100X, 20%) as negative and positive controls. (a) P-(*t*-Bu-DMEA-Caf30%)MA, (b) P-(*t*-Bu-DMEA-Caf10%)MA, and (c) P-(*t*-Bu-DMEA)MA. Data are represented as mean ± SEM (*n* = 3). One-way ANOVA was performed followed by Tukey’s test (***P < 0.001).

We then evaluated the hemocompatibility of the caffeine-cationic copolymers by determining the hemolytic effect on erythrocytes. For the assay, sheep erythrocytes were incubated at 37 °C for 1 h with different polymer concentrations (1–1000 μg/mL) and the absorbance of released hemoglobin was measured at 540 nm. We observed that the copolymer series P-(ab-Caf)MA and P-(*t-*Bu-DMEA-Caf)MA presented a low hemolytic effect (Figures 4 and S43). Interestingly, the cationic polymers bearing primary ammonium groups and caffeine (P-(ab-Caf)MA) presented a low hemolytic effect (<10% hemolysis) even at 50% of caffeine content (Figure S43). These results are important because usually a hydrophobic content >30% leads to increased hemolysis and cytotoxicity^15^ and the use of caffeine that contains a purine core could be an approach to access antimicrobial polymers with a lower hemolytic activity while preserving the antimicrobial activity. Then, hemolysis was evaluated for the quaternary ammonium copolymers with caffeine using the same methodology. Interestingly, the copolymers presented low hemolysis values of 3.5–3.7% even if the hydrophobic content was increased using caffeine. Indeed, the anti-infective copolymers P-(*t*-Bu-DMEA-Caf10%)MA and P-(*t*-Bu-DMEA)MA showed hemolysis values of 3.5 ± 1.2% and 3.7 ± 0.4%, respectively at a polymer concentration of 1000 μg/mL (Figure 4). These results demonstrated hemocompatibility even at high hydrophobic content provided by the caffeine side chain.

In summary, the copolymers bearing cationic or zwitterionic side chains with caffeine were prepared successfully through free radical polymerization and tested against the Gram-negative *E. coli* and the Gram-positive *S. aureus*. As expected, it was observed that cationic side chains were necessary to present activity against bacteria as primary ammonium (P-(ab-Caf)MA) and quaternary ammonium copolymers (P-(*t*-Bu-DMEA-Caf)MA) inhibited bacteria growth. In this sense, primary ammonium copolymers inhibited the growth exclusively for Gram-negative, while quaternary ammonium copolymers presented a broad spectrum of action. It was also found that finely tuning the hydrophobic ratio allowed optimization of antimicrobial polymers, consistent with other work on selectivity.^49^ Interestingly, after we evaluated the cytotoxicity and hemolysis, it was observed that the copolymers with the best MIC value, were also the ones that presented good cell viability (>80%) and low hemolysis (<10%). The copolymer P-(ab-Caf 30%)MA was the optimal polymer among those tested with MIC = 26 ± 2 μg/mL and cell viability dropping below 80% at 250 μg/mL. The best copolymers bearing quaternary ammonium had 10% or 0% caffeine (P-(*t*-Bu-DMEA-Caf 10%)MA and P-(*t*-Bu-DMEA)MA) with MIC = 15–60 μg/mL and cell viability > 80% even at 1000 μg/mL, as well as low hemolysis. It is important to note that P-(*t*-Bu-DMEA)MA is a quaternary ammonium homopolymer, and the antibacterial activity and low toxicity might be related to a hydrophilic polycation mechanism, rather than to membrane disruption, which might characterize the polymers having caffeine as a hydrophobic side chain.^22,50^ Therefore, the mechanism of these antimicrobial polymers needs further study. Collectively, the data shows that caffeine side chains helped to preserve antimicrobial activity and cell viability, as well as low hemolysis of copolymers.

## Conclusion

In this work, we prepared caffeine-derived methacrylate copolymers bearing cationic or zwitterionic side chains for antimicrobial purposes. These cationic copolymers presented a broad spectrum against Gram-positive and -negative bacteria. Moreover, the polymers possessed good biocompatibility as confirmed by MTT assay in a mice fibroblast cell line (NIH 3T3) and hemolysis assay. Therefore, the use of caffeine and *tert*-butyl-protected carboxybetaine as hydrophobic and cationic groups could help to prepare selective polymers against bacteria with low cytotoxic effects.

## Materials and Methods

### Materials

All the chemicals and solvents in this work were purchased from Sigma-Aldrich and TCI and, unless otherwise described, were used without any purification. AIBN was recrystallized from acetone before use. DMSO was dried using molecular sieves and kept under an inert atmosphere. Milli-Q water was obtained from a Purelab Prima, ELGA. Sterile U-bottom 96-well plates (Falcon) and Mueller Hinton Broth (NutriSelect Plus, Sigma-Aldrich) were used for all broth microdilution tests, along with the following bacteria strains: *Escherichia coli* (Migula) Castellani and Chalmers (ATCC27065, ATCC), *Staphylococcus aureus* strain Newman (donation from Dr. Robert Clubb, University of California, Los Angeles), and two clinical isolates of methicillin-resistant *Staphylococcus aureus* (donation from Dr. Shangxin Yang, University of California, Los Angeles). Phosphate buffer saline (PBS) (pH 7.4), Dulbecco’s Modified Eagle’s Medium (DMEM) cell culture media, and Pen Strep (Penicillin and Streptomycin) were procured from Gibco, ThermoFisher Scientific. Fetal bovine serum (FBS) was procured from Sigma-Aldrich. Trypsin was procured from MP Biomedicals. MTT (3-(4,5-dimethylthiazol-2-yl)-2,5-diphenyltetrazolium bromide) was procured from RPI (Research Product International Corp.). DMSO was procured from VWR chemicals.

### Methods

NMR spectra were obtained on a Bruker AV 400 MHz instrument (UCLA, CA, USA), and the data was analyzed using MestRenova v12 software. Size exclusion chromatography (SEC) measurements in the trifluoroethanol (TFE) phase were performed on a JASCO BS 4000-1 HPLC System, equipped with a UV-4075 UV/vis detector. The system also included a differential refractive index detector RI-4030 RI detector. Polymers were separated on a mixed-column system equipped with two PSS PFG columns (8 × 300 mm, 5 μm) at a flow rate of 0.5 mL/min. Column temperatures were held at 23 °C in TFE 20 mM NaTFA. Molar mass was calculated from a calibration curve of poly(methyl methacrylate). Size exclusion chromatography (SEC) measurements in DMF were performed on an Infinity 1260 II HPLC system from Agilent equipped with a diode array detector DAD from Wyatt technology. The system also included a multiangle light scattering detector MALS and differential refractive index detector dRI from Wyatt technology. Polymers were separated on two PLgel Mixed-D gel columns PL1110–6504 (300 × 7.5 mm) (exclusion limits from 200 to 400 000 Da) at a flow rate of 0.6 mL min^–1^. Column temperatures were held at 40 °C in DMF with LiBr (0.1 M). Molar masses were calculated from the d*n*/d*c* of the different polymers. Size exclusion chromatography (SEC) measurements in the aqueous phase were performed on a Waters Alliance HPLC System, 2695 Separation Module equipped with photodiode array detector 2998 PDA from Watters technology. The system also included a multiangle light scattering detector MALS and differential refractive index detector dRI from Wyatt technology. Polymers were separated on Tosoh TSKgel G3000PWXL (7 μm) + TSKgel G5000PWXL (10 μm) at a flow rate of 1.0 mL/min. Column temperatures were held at 23 °C in Milli-Q Water with 0.2 M NaNO3 + 0.1% TFA. Molar masses were calculated from the d*n*/d*c* of the different polymers.

#### Monomer Preparation

##### Synthesis of 2-(1,3-Dimethyl-2,6-dioxo-1,2,3,6-tetrahydro-7*H*-purin-7-yl)ethyl Methacrylate; Ethofylline-methacrylate (CafMA)

In a round-bottom flask etofylline 99% (1g, 4 mmol, 1 equiv) was dissolved in 15 mL of dry DCM, placed in an ice bath, and mixed with triethylamine 97% (Et_3_N 0.7 mL, 5 mmol, 1.1 equiv). Then, methacryloyl chloride 90% (0.6 mL, 5 mmol, 1.2 equiv) was added slowly. The reaction was stirred at 23 °C for 16 h. Upon completion, as attested by TLC (DCM:Acetone 8:2, Rf _product_ = 0.76, Rf _caffeine OH_ = 0.1), the solution was filtered, and the organic phase was dried using rotavapor. Then, the powder was resolubilized in 10 mL of DCM, placed in a separation funnel, and followed by the addition of 30 mL of water and 2 mL of HCl. The organic phase was separated, and the aqueous phase was extracted twice using 10 mL of DCM. The organic phase was dried using MgSO4, filtered, and dried in a rotavapor. The product was isolated as a slightly yellowish powder in a 56% yield.

^1^H NMR (400 MHz, DMSO-*d*_6_) δ (ppm): 8.12 (s, 1H CH), 5.95 (s, 1H, CH_2_), 5.65 (s, 1H, CH_2_), 4.57 (dd, *J* = 5.6, 4.2 Hz, 2H, CH_2_), 4.44 (dd, *J* = 5.7, 4.2 Hz, 2H, CH_2_), 3.42 (s, 3H, CH_3_), 3.22 (s, 3H, CH_3_), 1.80 (s, 3H, CH_3_). ^1^H NMR signals agreed with the previous report.^51^

^13^C NMR (101 MHz, DMSO) δ (ppm): 166.01 (CO), 154.50 (CO), 151.00 (CO), 148.45 (C), 143.06 (CH), 135.42 (C), 126.20 (CH_2_), 106.01 (C), 62.93 (CH_2_), 45.31 (CH_2_), 39.52 (CH_2_), 29.46 (CH_3_), 27.55 (CH_3_), 17.83 (CH_3_).

##### Synthesis of *tert*-Butyl (4-Methacrylamidobutyl)carbamate (Boc-ab-MA)

In a round-bottom flask *tert*-butyl(4-aminobutyl) carbamate 95% (5 mL, 24.82 mmol, 1 equiv) and Et_3_N 97% (4.28 mL, 29.79 mmol, 1.2 equiv) were dissolved in 41 mL of DCM and placed them in an ice bath. Then, methacryloyl chloride 90% (3.34 mL, 29.79 mmol, 1.2 equiv) was added slowly. The reaction was stirred reaching 23 °C conditions for 16 h. Then, the solution was washed with 10 mL of HCl 3 M and 50 mL of water three times. The organic phase was dried with magnesium sulfate, filtered off and the solvent was evaporated using the rotavapor. The solid was washed with cyclohexane and isolated in a 65% yield as a white powder.

^1^H NMR (400 MHz, DMSO-*d*_6_) δ (ppm): 7.88 (s, 1H, NH), 6.78 (s, 1H, NH), 5.61 (s, 2H, CH_2_), 5.29 (s, 1H, CH_2_), 3.07 (q, *J* = 6.3 Hz, 2H, CH_2_), 2.89 (q, *J* = 6.3 Hz, 2H, CH_2_), 1.83 (s, 3H, CH_3_), 1.37 (s, 13H, 2CH_2_ + 3CH_3_).

^13^C NMR (101 MHz, DMSO) δ (ppm): 167.34 (CO), 155.57 (CO), 140.13 (C), 118.63 (CH_2_), 77.32 (C), 39.52 (DMSO+CH_2_), 38.57 (CH_2_), 28.27 (CH_3_), 27.03 (CH_2_), 26.52 (CH_2_), 18.68(CH_3_).

MS ESI^+^*m*/*z* [M + Na]^+^ = 279.16 (expected *m*/*z* (C_13_H_24_N_2_O_3_Na) = 279.17).

##### Synthesis of 2-(*tert*-Butoxy)-*N*-(2-(methacryloyloxy)ethyl)-*N,N*-dimethyl-2-oxoethan-1-aminium (*t*-Bu-DMEA-MA)

The monomer was prepared as reported elsewhere with slight modification.^52^ In a round-bottom flask 2-(dimethylamino) ethyl methacrylate 97% (5.15 mL, 30.85 mmol, 1 equiv) and *tert*-butyl 2-bromoacetate 97% (5.64 mL, 37 mmol, 1.2 equiv) were mixed in 50 mL of ACN. The mixture was stirred at 50 °C for 16 h. Then the solvent was evaporated. The crude oil was mixed with 15 mL of DCM and precipitated in 200 mL of Et_2_O twice. The powder was isolated as a white powder in an 83% yield.

^1^H NMR (400 MHz, DMSO-*d*_6_) δ (ppm): 6.08–6.06 (m, 1H, CH_2_), 5.76 (t, *J* = 1.6 Hz, 1H, CH_2_), 4.55–4.52 (m, 2H, CH_2_), 4.50 (s, 2H, CH_2_), 3.97–3.91 (m, 2H, CH_2_), 3.29 (s, 6H, CH_3_), 1.90 (s, 3H, CH_3_), 1.46 (s, 9H, 3CH_3_). ^1^H NMR signals agreed with the previous report.^52^

##### Synthesis of Lysine Methacrylate (Lys-MA)

The monomer was prepared as reported elsewhere with slight modification.^53^ In a round-bottom flask, CbzLys-OH 99% (2 g, 7.13 mmol, 1 equiv), methacryloyl chloride 90% (1.6 mL, 14.2 mmol, 2 equiv), and NaCO_3_ 97% (1.24 g, 14.2 mmol, 2 equiv) were dissolved in 5 mL of water and 24 mL of THF. The mixture was stirred in an ice bath for 30 min and then for 3 h at 23 °C. Then, 150 mL of water and 10 mL of 3 M HCl were added and extracted with DCM 50 mL three times. The organic phase was dried with magnesium sulfate, filtered off, and then, 10 μL of MEHQ (20 mg/mL) was added to prevent autopolymerization, and the solvent was evaporated in the rotavapor. The crude oil was then solubilized in TFA 10 mL and deprotected with HBr 33% (2.5 mL, 14.2 mmol, 2 equiv) for 4 h. Then it was precipitated in Et_2_O 200 mL and kept in the freezer at −20 C for 3h. The mixture was centrifuged at 4000 rpm for 4 min. The supernatant was discarded, and the powder was resolubilized in 5 mL of ethanol followed by the addition of triethylamine in excess. The mixture was centrifuged at 400 rpm for 4 min. The solid was dried at a high vacuum affording a yellowish solid in 30% yield.

^1^H NMR (400 MHz, DMSO-*d*_6_) δ (ppm): 8.38 (s, 3H, NH_3_^+^), 7.96 (s, 1H, NH), 5.63 (s, 1H, CH_2_), 5.30 (s, 1H, CH_2_), 3.13–3.02 (m, 2H, CH_2_), 1.83 (s, 3H, CH_3_), 1.77 (m, 2H, CH_2_), 1.49–1.28 (m, 4H, 2CH_2_). ^1^H NMR signals agreed with the previous report.^53^

#### Polymer Preparation

##### Synthesis of Poly(etofylline)methacrylate (CafMA)

AIBN (90 mg, 6.6 μmol, 1 equiv) was dissolved in 220 μL of DMSO (dried with molecular sieves) and transferred to a dried Schlenk tube, and CafMA (5 mg, 0.2 mmol, 30 equiv) was added. The mixture was degassed four times using the freeze–thawing technique. The reaction was stirred at 75 °C for 1.5 h after confirming the absence of monomer. The polymer was precipitated in 20 mL of Et_2_O and centrifuged at 4000 rpm for 5 min, repeating this action three times. The polymer was dried *in vacuo* and recovered as a white powder (yield 75%). *M*_*n*_ = 25.4 kg/mol *Đ* = 2.77.

^1^H NMR (400 MHz, Chloroform-*d*) δ (ppm): 7.84 (br, 1H, CH), 4.61 (br, 2H, CH_2_), 4.29 (br, 2H, CH_2_), 3.54 (br, 3H, CH_3_), 3.33 (br, 3H, CH_3_), 1.76 (br, 2H, CH_2_), 0.58 (br, 3H, CH_3_).

##### Synthesis of Poly[(*tert*-butyl(4-methacrylamidobutyl)carbamate)-(etofylline-methacrylate)]; P(Boc-ab-Caf50%)MA

The monomer Boc-ab-MA (88.6 mg, 3.42 × 10^–4^ mol, 15 equiv) was transferred to a Schlenk vessel followed by the addition of CafMA (100 mg, 3.42 × 10^–4^ mol, 15 equiv) in 530 μL of DMF, the monomers were solubilized with gentle heat, and then 72.4 μL of AIBN (2.28 × 10^–5^ mol, 1 equiv) was added (solution of 20.75 mg in 400 μL of DMSO). The mixture was degassed four to five times using the freeze–thawing technique. The reaction was stirred at 75 °C overnight for 16 h. The polymers were precipitated in 25 mL of Et_2_O, and the solid was resolubilized in appoximately 1 mL DCM and reprecipitated in 25 mL of Et_2_O. The solid was dried *in vacuo* and isolated as a yellowish powder in a 69% yield. *M*_*n*_ = 27.7 kg/mol *Đ* = 1.75.

^1^H NMR (400 MHz, DMSO-*d*_6_) δ (ppm): 8.05 (br, 1H, CH), 7.22 (br, 1H, NH), 6.74 (br, 1H, NH), 4.52 (br, 2H, CH_2_), 4.15 (br, 2H, CH_2_), 3.32 (br, 3H, H_2_O+CH_3_), 3.14 (br, 3H, CH_3_), 2.89 (br, 4H, 2CH_2_), 1.35 (br, 20H, 4CH_2_, 3CH_3_), 0.49 (br, 6H, 2CH_3_).

The caffeine content was calculated by correlating the protons in methyl groups of the polymer backbone (0.49 ppm, CH_3_) and the protons of the caffeine side chain (8.05 ppm, CH) using the following equation:
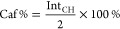


##### Synthesis of Poly[(*tert*-butyl(4-methacrylamidobutyl)carbamate)-(etofylline-methacrylate)]; P(Boc-ab-Caf30%)MA

The copolymer was prepared similarly to P(Boc-ab-Caf50%)MA varying the equivalent ratio of Boc-ab-MA and CafMA. Upon isolation, the copolymer was obtained as a yellowish powder in a 73% yield. *M*_*n*_ = 21.8 kg/mol *Đ* = 1.74.

^1^H NMR (400 MHz, DMSO-*d*_6_) δ (ppm): 8.05 (br, 0.7H, CH), 7.22 (br, 1H, NH), 6.74 (br, 1H, NH), 4.52 (br, 1H, CH_2_), 4.15 (br, 1H, CH_2_), 3.32 (br, 3H, H_2_O+CH_3_), 3.14 (br, 2.6H, CH_3_), 2.89 (br, 4H, 2CH_2_), 1.35 (br, 20H, 4CH_2_, 3CH_3_), 0.49 (br, 6H, 2CH_3_).

##### Synthesis of Poly[(*tert*-butyl(4-methacrylamidobutyl)carbamate)-(etofylline-methacrylate)]; P(Boc-ab-Caf10%)MA

The copolymer was prepared similarly to P(Boc-ab-Caf50%)MA varying the equivalent ratio of Boc-ab-MA and CafMA. Upon isolation, the copolymer was obtained as a yellowish powder in a 68% yield. *M*_*n*_ = 25.8 kg/mol *Đ* = 1.50.

^1^H NMR (400 MHz, DMSO-*d*_6_) δ (ppm): 8.11 (br, 0.12H, CH), 7.21 (br, 1.6H, NH), 6.75 (br, 1H, NH), 4.52 (br, 0.29H, CH_2_), 4.15 (br, 0.29H, CH_2_), 3.32 (br, 0.29H, H_2_O+CH_3_), 3.14 (br, 1.02H, CH_3_), 2.89 (br, 4H, 2CH_2_), 1.36 (br, 20H, 4CH_2_, 3CH_3_), 0.49 (br, 6H, 2CH_3_).

##### Synthesis of Poly(*tert*-butyl(4-methacrylamidobutyl)carbamate); P(Boc-ab)MA

The copolymer was prepared similarly to P(Boc-ab-Caf50%)MA using only Boc-ab-MA. Upon isolation, the copolymer was obtained as a yellowish powder in a 67% yield. *M*_*n*_ = 23.2 kg/mol *Đ* = 1.52.

^1^H NMR (400 MHz, DMSO-*d*_6_) δ (ppm): 7.20 (br, 1H, NH), 6.75 (br, 1H, NH), 2.89 (br, 4H, 2CH_2_), 1.36 (br, 17H, 4CH_2_, 3CH_3_), 0.49 (br, 3H, CH_3_).

##### Synthesis of Poly[(*N*-(4-aminobutyl)-methacrylamide)-(etofylline-methacrylate)]; P(ab-Caf50%)MA

An amount of 100 mg of P(Boc-ab-Caf50%)MA polymer was dissolved in 1 mL of TFA and stirred for 4 h at 23 °C. Then the product was precipitated in 25 mL of Et_2_O, centrifuged at 4500 rpm for 5 min, reprecipitated, centrifuged, dried in vacuo, and dialyzed with deionized water (MWCO 3.5 kDa). Upon isolation, the copolymer was isolated as a yellowish powder in a 69% yield. No SEC data was obtained due to poor solubility under the analytical conditions.

^1^H NMR (400 MHz, DMSO-*d*_6_) δ (ppm): 8.06 (br, 1.1H, CH), 7.38 (br, 1.5H, NH), 4.52 (br, 1.87H, CH_2_), 4.14 (br, 1.87H, CH_2_), 3.32 (br, 6H, H_2_O+3CH_2_), 2.79 (br, 2.2H, CH_2_), 1.51 (br, 5H, 4CH_2_), 0.53 (br, 6H, 2CH_3_).

##### Synthesis of Poly[(*N*-(4-aminobutyl)-methacrylamide)-(etofylline-methacrylate)]; P(ab-Caf30%)MA

The copolymer was deprotected similarly as described for P(ab-Caf50%)MA. Upon isolation, the copolymer was isolated as a yellowish powder in a 70% yield. No SEC data was obtained due to poor solubility under the analytical conditions.

^1^H NMR (400 MHz, DMSO-*d*_6_) δ (ppm): 8.22 (br, 0.67H, CH), 7.38 (br, 1.16H, NH), 4.57 (br, 1.27H, CH_2_), 4.18 (br, 1.27H, CH_2_), 3.32 (br, 6H, H_2_O+3CH_2_), 2.63 (br, 2.2H, CH_2_), 1.52 (br, 8.39H, 4CH_2_), 0.70 (br, 6H, 2CH_3_).

##### Synthesis of Poly[(*N*-(4-aminobutyl)-methacrylamide)-(etofylline-methacrylate)]; P(ab-Caf10%)MA

The copolymer was deprotected similarly as described for P(ab-Caf50%)MA. Upon isolation, the copolymer was isolated as a yellowish powder in an 81% yield. No SEC data was obtained due to poor solubility under the analytical conditions.

^1^H NMR (400 MHz, DMSO-*d*_6_) δ (ppm): 8.22 (br, 0.19H, CH), 7.38 (br, 0.55H, NH), 4.57 (br, 0.33H, CH_2_), 4.18 (br, 0.33H, CH_2_), 3.32 (br, 6H, H_2_O+3CH_2_), 2.63 (br, 2.82H, CH_2_), 1.54 (br, 7.65H, 4CH_2_), 0.70 (br, 6H, 2CH_3_).

##### Synthesis of Poly(*N*-(4-aminobutyl)-methacrylamide); P(ab-MA)

The copolymer was deprotected similarly as described for P(ab-Caf50%)MA. Upon isolation, the copolymer was isolated as a yellowish powder in a 73% yield. No SEC data was obtained due to poor solubility under the analytical conditions.

^1^H NMR (400 MHz, DMSO-*d*_6_) δ (ppm): 4.44 (br, 1.29H, CH_2_), 2.94 (br, 2.55H, CH_2_), 2.76 (br, 2.32H, CH_2_), 1.55 (br, 5.08H, 4CH_2_), 0.80 (br, 3H, 2CH_3_).

##### Synthesis of Poly[(2-(*tert*-butoxy)-*N*-(2-(methacryloyloxy)ethyl)-*N,N*-dimethyl-2-oxoethan-1-aminium)-(etofylline-methacrylate)]; P(*t*-Bu-DMEA-Caf30%)MA

The monomer *t*-Bu-DMEA-MA (170.4 mg, 4.79 × 10^–4^ mol, 21 equiv) was transferred to a Schlenk tube followed by the addition CafMA (60 mg, 2.05 × 10^–4^ mol, 9 equiv) in 530 μL of DMSO. The monomers were solubilized with gentle heat and then 35.4 μL of AIBN (2.28 × 10^–5^ mol, 1 equiv) was added (AIBN solution = 21.19 mg in 200 μL of DMSO). The mixture was degassed four to five times using the freeze–thawing technique. The reaction was stirred at 75 °C for 16 h. The polymer was then precipitated in 20 mL of Et_2_O and centrifuged at 4500 rpm for 5 min. The solid was resolubilized in DCM 1 mL, reprecipitated in 25 mL Et_2_O, centrifuged, and dried in vacuo. Upon isolation, the copolymer was obtained as a yellowish powder in an 80% yield. No SEC data was obtained due to poor solubility under the analytical conditions.

^1^H NMR (400 MHz, DMSO) δ (ppm): 8.36 (br, 0.52H, CH), 4.71 (br, 7.68H, 5CH_2_), 3.36 (br, 7.68H, 4CH_2_), 1.47 (br, 16.94, 4CH_2_ + 3CH_3_), 0.83 (br, 6H, 2CH_3_).

##### Synthesis of Poly[(2-(*tert*-butoxy)-*N*-(2-(methacryloyloxy)ethyl)-*N,N*-dimethyl-2-oxoethan-1-aminium)-(etofylline-methacrylate)]; P(*t*-Bu-DMEA-Caf10%)MA

The copolymer was prepared similarly to P(Boc-ab-Caf30%)MA varying the equivalent ratio of *t*-Bu-DMEA-MA and CafMA. Upon isolation, the copolymer was obtained as a yellowish powder in an 83% yield. *M*_*n*_ = 21.1 kg/mol *Đ* = 2.0.3.

^1^H NMR (400 MHz, DMSO) δ (ppm): 8.48 (br, 0.16H, CH), 4.71 (br, 9.92H, 5CH_2_), 3.36 (br, 7.92H, 4CH_2_), 1.47 (br, 16.41, 4CH_2_ + 3CH_3_), 0.83 (br, 6H, 2CH_3_).

##### Synthesis of Poly(2-(*tert*-butoxy)-*N*-(2-(methacryloyloxy)ethyl)-*N,N*-dimethyl-2-oxoethan-1-aminium); P(*t*-Bu-DMEA)MA

The copolymer was prepared similarly to P(Boc-ab-Caf30%)MA using only *t*-Bu-DMEA-MA. Upon isolation, the copolymer was obtained as a yellowish powder in a 92% yield. *M*_*n*_ = 32.0 kg/mol *Đ* = 2.06.

^1^H NMR (400 MHz, DMSO) δ (ppm): 8.48 (br, 0.16H, CH), 4.71 (br, 9.92H, 5CH_2_), 3.36 (br, 7.92H, 4CH_2_), 1.47 (br, 16.41, 4CH_2_ + 3CH_3_), 0.83 (br, 6H, 2CH_3_).

##### Synthesis of Poly[(2-((2-(methacryloyloxy)ethyl)dimethylammonio)acetate)-(etofylline-methacrylate)]; P(DMEA-Caf30%)MA

Deprotection was performed by dissolving 100 mg of polymer in 1 mL of TFA. The solution was stirred for 4 h at 23 °C and then the polymer was precipitated in 20 mL Et_2_O, centrifuged at 4500 rpm for 5 min, reprecipitated, centrifuged, and dialyzed in water (MWCO = 3.5 kDa). The polymer was freeze-dried and obtained as a yellowish powder in a 91% yield. *M*_*n*_ = 16.0 kg/mol *Đ* = 2.24.

^1^H NMR (400 MHz, D_2_O) δ (ppm): 8.13 (br, 0.46H, CH), 4.25 (br, 6.41H, 5CH_2_), 3.38 (br, 8.04H, 4CH_2_), 1.94 (br, 3.75H, 4CH_2_), 0.68 (br, 6H, 2CH_3_).

##### Synthesis of Poly[(2-((2-(methacryloyloxy)ethyl)dimethylammonio)acetate)-(etofylline-methacrylate)]; P(DMEA-Caf10%)MA

The copolymer was deprotected similarly as described for P(DMEA-Caf30%)MA. Upon isolation, the copolymer was isolated as a yellowish powder in an 86% yield. *M*_*n*_ = 26.7 kg/mol *Đ* = 2.53.

^1^H NMR (400 MHz, D_2_O) δ (ppm): 8.15 (br, 0.22H, CH), 4.21 (br, 8.57H, 5CH_2_), 3.38 (br, 8.49H, 4CH_2_), 1.96 (br, 2.49H, 4CH_2_), 0.99 (br, 6H, 2CH_3_).

##### Synthesis of Poly(2-((2-(methacryloyloxy)ethyl)dimethylammonio)acetate); P(DMEA)MA

The copolymer was deprotected similarly as described for P(DMEA-Caf30%)MA. Upon isolation, the copolymer was isolated as a yellowish powder in a 91% yield. *M*_*n*_ = 27.7 kg/mol *Đ* = 3.24.

^1^H NMR (400 MHz, D_2_O) δ (ppm): 4.26 (br, 5.45H, 3CH_2_), 3.38 (br, 5.46H, 2CH_2_), 2.01 (br, 2.12H, 2CH_2_), 0.97 (br, 3H, CH_3_).

##### Synthesis of Poly((lysine-methacrylate)-(etofylline-methacrylate)); P(Lys-Caf50%)MA

The monomer LysMA (55.5 mg, 2.57 × 10^–4^ mol, 15 equiv) was transferred to a Schenck vessel followed by the addition CafMA (75 mg, 2.57 × 10^–4^ mol, 15 equiv) in 0.6 mL of DMSO and 0.2 mL of HCl 6 M. The monomers were solubilized with gentle heat, and then 35.8 μL of AIBN (1.71 × 10^–5^ mol, 1 equiv) was added (solubilizing 16.04 mg in 200 μL of DMSO). The mixture was degassed four times using the freeze–thawing technique. The reaction was stirred at 75 °C for 16 h. The polymers were precipitated in 20 mL of Et_2_O, centrifuged at 4500 rpm for 5 min, reprecipitated, centrifuged, and dialyzed in deionized water (MWCO 3.5 kDa). The polymer was obtained as a yellowish powder in a 4% yield. No SEC data was obtained due to poor solubility under the analytical conditions.

^1^H NMR (400 MHz, DMSO-*d*_6_) δ (ppm): 8.10 (br, 1.19H, CH), 4.58 (br, 6.09H, 3CH_2_), 3.18 (br, 7.42H, 2CH_2_), 1.40 (br, 6.90H, 6CH_2_), 0.44 (br, 6H, 2CH_3_).

##### Synthesis of Poly((lysine-methacrylate)-(etofylline-methacrylate)); P(Lys-Caf30%)MA

The copolymer was prepared similarly to P(Lys-Caf50%)MA varying the equivalent ratio of LysMA and CafMA. Upon isolation, the copolymer was obtained as a yellowish powder in a 39% yield. *M*_*n*_ = 18.0 kg/mol *Đ* = 2.24.

^1^H NMR (400 MHz, DMSO-*d*_6_ + 15% TFA) δ (ppm): 8.24 (br, 1.95H, NH+NH_2_), 8.10 (br, 0.76H, CH), 4.58 (br, 3.08H, 2CH_2_), 3.87 (br, 1.24, CH), 3.12 (br, 5.81H, 2CH_2_), 1.77 (br, 2.75H, 2CH_2_), 1.39 (br, 5.06H, 4CH_2_), 0.50 (br, 6H, 2CH_3_).

##### Synthesis of Poly((lysine-methacrylate)-(etofylline-methacrylate); P(Lys-Caf10%)MA

The copolymer was prepared similarly to P(Lys-Caf50%)MA varying the equivalent ratio of LysMA and CafMA. Upon isolation, the copolymer was obtained as a yellowish powder in a 38% yield. *M*_*n*_ = 17.8 kg/mol *Đ* = 2.25.

^1^H NMR (400 MHz, DMSO-*d*_6_ + 15% TFA) δ (ppm): 8.28 (br, 2.47H, NH+NH_2_), 8.10 (br, 0.20H, CH), 4.58 (br, 0.37H, 2CH_2_), 3.86 (br, 1.30, CH), 2.90 (br, 4.88H, 2CH_2_), 1.77 (br, 4.73H, 2CH_2_), 1.38 (br, 7.74H, 4CH_2_), 0.80 (br, 6H, 2CH_3_).

##### Synthesis of Poly(lysine-methacrylate); P(Lys)MA

The copolymer was prepared similarly to P(Lys-Caf50%)MA using only LysMA. Upon isolation, the copolymer was obtained as a yellowish powder in a 17% yield. *M*_*n*_ = 25.2 kg/mol *Đ* = 2.28.

^1^H NMR (400 MHz, DMSO-*d*_6_ + 15% TFA) δ (ppm): 8.30 (br, 1.33H, NH+NH_2_), 3.88 (br, 1.26, CH), 1.77 (br, 3.39H, 2CH_2_), 1.38 (br, 4.96H, 4CH_2_), 0.81 (br, 3H, CH_3_).

##### Synthesis of Poly[(2-(*tert*-butoxy)-*N*-(2-(methacryloyloxy)ethyl)-*N,N*-dimethyl-2-oxoethan-1-aminium)-(etofylline-methacrylate)]; P(*t*-Bu-DMEA-Caf30%)MA DP30

The monomer *t*-Bu-DMEA-MA (204.5 mg, 5.75 × 10^–4^ mol, 21 equiv) was transferred to a Schlenk tube followed by the addition CafMA (72 mg, 2.46 × 10^–4^ mol, 9 equiv) in 630 μL of DMSO. The monomers were solubilized with gentle heat, and then AIBN (1.12 mg, 6.84 × 10^–6^ mol, 0.25 equiv) and 4-cyano-4-(phenyl carbonothioyllthio)pentanoic acid 99% (CTA, 7.72 mg, 2.74 × 10^–5^ mol, 1 equiv) were added. The mixture was degassed four times using the freeze–thawing technique. The reaction was stirred at 75 °C for 16 h. The polymers were precipitated in 25 mL of Et_2_O and centrifuged at 4500 rpm for 5 min. The solid was resolubilized in DCM 1 mL, reprecipitated in 25 mL of Et_2_O, centrifuged, and dried in vacuo. Upon isolation, the copolymer was obtained as a yellowish powder in a 70% yield. *M*_*n*_ = 6.4 kg/mol *Đ* = 1.16.

^1^H NMR (400 MHz, DMSO) δ (ppm): 8.30 (br, 11.88H, CH), 7.86–7.46 (m, 5H, 5CH), 4.35 (m, 383H, 8CH_2_), 3.68–3.40 (m, 274H, 4CH_3_), 1.76 (br, 116H, 3CH_2_), 1.47 (br, 312H, 3CH_3_), 0.78 (br, 104H, 2CH_3_).

The polymerization degree (DP) was calculated using the methyl group in the polymer backbone (signal 0.78 ppm, CH_3_) according to the following equation:
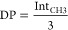


The caffeine content was calculated by correlating the polymerization degree and the protons of the caffeine side chain (8.30 ppm, CH) using the following equation:
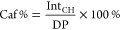


##### Synthesis of Poly[(2-(*tert*-butoxy)-*N*-(2-(methacryloyloxy)ethyl)-*N,N*-dimethyl-2-oxoethan-1-aminium)-(etofylline-methacrylate)]; P(*t*-Bu-DMEA-Caf10%)MA DP30

The copolymer was prepared similarly to P(*t*-Bu-DMEA-Caf30%)MA DP30 varying the equivalent ratio of *t-*Bu-DMEA-MA and CafMA. Upon isolation, the copolymer was obtained as a yellowish powder in a 71% yield. *M*_*n*_ = 6.3 kg/mol *Đ* = 1.06.

^1^H NMR (400 MHz, DMSO) δ (ppm): 8.30 (br, 3.74H, CH), 7.86–7.46 (m, 5H, 5CH), 4.35 (m, 294H, 8CH_2_), 3.76–3.40 (m, 189H, 4CH_3_), 1.82 (br, 94.46H, 3CH_2_), 1.47 (br, 246H, 3CH_3_), 0.82 (br, 87.36H, 2CH_3_).

##### Synthesis of Poly(2-(*tert*-butoxy)-*N*-(2-(methacryloyloxy)ethyl)-*N,N*-dimethyl-2-oxoethan-1-aminium); P(*t*-Bu-DMEA)MA DP30

The copolymer was prepared similarly to P(*t*-Bu-DMEA-Caf30%)MA DP30 using only *t*-Bu-DMEA-MA. Upon isolation, the copolymer was obtained as a yellowish powder in a 76% yield. *M*_*n*_ = 4.7 kg/mol *Đ* = 1.03.

^1^H NMR (400 MHz, DMSO) δ (ppm): 7.85–7.46 (m, 5H, 5CH), 4.42 (m, 324H, 5CH_2_), 3.74–3.41 (m, 233H, 2CH_3_), 1.88 (br, 122H, 3CH_2_), 1.49 (br, 256H, 3CH_3_), 0.99 (br, 90H, 2CH_3_).

##### Synthesis of Poly[(2-((2-(methacryloyloxy)ethyl)dimethylammonio)acetate)-(etofylline-methacrylate))]; P(DMEA-Caf30%)MA DP30

Deprotection was performed by dissolving 100 mg of polymer in 1 mL of TFA. The solution was stirred for 4 h at 23 °C, and then the polymer was precipitated in 20 mL Et_2_O, centrifuged at 4500 rpm for 5 min, reprecipitated, centrifuged, and dialyzed in water (MWCO = 3.5 kDa). The polymer was freeze-dried and obtained as a yellowish powder in a 72% yield. No SEC data was obtained due to poor solubility under the analytical conditions.

^1^H NMR (400 MHz, D_2_O) δ (ppm): 8.14 (br, 10.06H, CH), 7.91–7.49 (m, 5H, 5CH), 4.45–3.99 (m, 173H, 8CH_2_), 3.52–3.35 (m, 221H, 4CH_2_), 1.86 (br, 92H, 3CH_2_), 0.90 (br, 108H, 2CH_3_).

##### Synthesis of Poly[(2-((2-(methacryloyloxy)ethyl)dimethylammonio)acetate)-(etofylline-methacrylate))]; P(DMEA-Caf10%)MA DP30

The copolymer was deprotected similarly as described for P(DMEA-Caf30%)MA DP30. Upon isolation, the copolymer was isolated as a yellowish powder in a 73% yield. *M*_*n*_ = 4.2 kg/mol *Đ* = 1.16.

^1^H NMR (400 MHz, D_2_O) δ (ppm): 8.18 (br, 2.69H, CH), 7.96–7.49 (m, 5H, 5CH), 4.44–4.00 (m, 204H, 8CH_2_), 3.52–3.35 (m, 217H, 4CH_2_), 2.02 (br, 74H, 3CH_2_), 1.18 (br, 94H, 2CH_3_).

##### Synthesis of Poly(2-((2-(methacryloyloxy)ethyl)dimethylammonio)acetate); P(DMEA)MA DP30

The copolymer was deprotected similarly as described for P(DMEA-Caf30%)MA DP30. Upon isolation, the copolymer was isolated as a yellowish powder in a 74% yield. *M*_*n*_ = 5.1 kg/mol *Đ* = 1.18.

^1^H NMR (400 MHz, D_2_O) δ (ppm): 7.98–7.53 (m, 5H, 5CH), 4.48–4.00 (m, 151H, 5CH_2_), 3.52–3.35 (m, 133H, 4CH_2_), 2.03 (br, 68H, 3CH_2_), 1.16 (br, 96H, 2CH_3_).

##### Minimum Inhibitory Concentration

Microdilution methodology was adapted from previously reported protocols.^54^ An amount of 50 μL of sterile Mueller Hinton Broth was added to every well in a 96-well plate, except for the last column, where 100 μL was added. Copolymers and antibiotics were resuspended in Milli-Q water to a concentration four times greater than the highest amount to be tested. 50 μL of the copolymers or antibiotics to be assayed was added to the first column, then 2-fold serial dilutions were carried out across the next nine wells in the row.

Strains to be tested were streaked on Mueller Hinton agar and incubated overnight at 37 °C. Single colonies were picked and added to Mueller Hinton broth to achieve an optical density equal to McFarland standard 0.5, then diluted 100-fold. A volume of 50 μL of the bacterial suspension was added to each well containing diluted copolymer or antibiotic, along with one column containing no antimicrobial agent to serve as growth control. The final column containing only 100 μL of broth was used to assess sterility. Covered plates were incubated without shaking at 37 °C for 24 h before visually assessing each well for growth.

##### Hemolysis Assay

To determine the hemolytic effects of the copolymers prepared the methodology was performed following a previous report.^55^ Sheep red blood cells (RBCs) from Innovative Research (lot 39841, 1 mL, 100%) were resuspended in 3 mL of PBS (1×, pH = 7.4) and centrifuged at 800*g* for 5 min, and the level of the supernatant was marked. The supernatant was discarded and filled out with more PBS. This washing step was performed 3–5 times. Then, the RBCs were resuspended in 47 mL of 1× PBS to obtain a 2% suspension (dilution 1:50). In an Eppendorf tube, 190 μL of RBCs was placed followed by the addition of 10 μL of polymer solution that 20-fold concentrated. Here, 1× PBS was used as the negative control and Triton X-100 (20% in PBS) was used as a positive control. The samples were incubated at 37 °C for 1 h. Then, the samples were centrifuged at 800*g* for 5 min. From the centrifuged samples, 100 μL of supernatant was placed in a 96-well plate and the absorbance was measured at 540 nm. To determine the hemolysis percentage, we use the following equation:
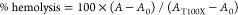
where *A* is the absorbance reading of the sample well, *A*_0_ is the negative hemolysis control (PBS 1×), and *A*_T100X_ is the positive hemolysis control (Triton X-100, 20%).

#### Biocompatibility

##### Cell Lines and Maintenance

The mouse embryonic fibroblasts, NIH 3T3, were cultured in Dulbecco’s Modified Eagle’s Medium (DMEM) media supplemented with 10% FBS (v/v), 1% Pen Strep, and antibiotics at 37 °C in a standard humidified atmosphere containing 5% carbon dioxide (CO_2_).

##### Methodology

The biocompatibility studies of the copolymers P(*t*-Bu-DMEA-Caf)MA and P(ab-Caf)MA were evaluated for 24 h in mouse embryonic fibroblasts, NIH 3T3, by performing the colorimetric MTT assay. For this assay, 0.7 × 10^4^ cells/well were seeded in 96-well plates and incubated overnight. The next day, the cells were treated with different concentrations of the polymer (1–1000 μg/mL) and incubated for 24 h. After the incubation, the cell culture media was replaced with the serum-free media containing MTT reagent (5 mg/10 mL) and incubated at 37 °C for 3 h. DMSO, a solubilizing agent, was added to dissolve the formazan crystals and the absorbance was measured at 570 nm using a plate reader SpectraMax iD3Multi-Mode Microplate Reader from Molecular Devices (UCLA, CA, USA).

##### Statistical Analysis

All experimental values are reported as the average ± standard deviation. Prism 9 software was used to analyze the results including the ANOVA and Turkey’s test. Results were considered significantly different if *p* < 0.05 (*); results are also reported with *p* < 0.01 (**), *p* < 0.001 (***), and *p* < 0.0001 (****).

## Supporting Information Available

Information related to NMR, SEC, and biological assays (PDF)
