# Supplementary material for: Caffeine and Cationic Copolymers with Antimicrobial Properties
Source: ACS Bio Med Chem Au. 2023 Feb 13;3(2):189–200. doi: 10.1021/acsbiomedchemau.2c00077 (PMC10119941; doi:10.1021/acsbiomedchemau.2c00077)
Supplement: Supplementary file 1 — bg2c00077_si_001.pdf [file bg2c00077_si_001.pdf]

SUPPORTING INFORMATION

Caffeine and cationic copolymers with antimicrobial properties

Pedro Salas-Ambrosio,[1] Shelby Vexler,[1] Rajalakshmi P S,[1] Irene A. Chen,[2] Heather Maynard.[1*]

[1] Department of Chemistry and Biochemistry and California Nano Systems Institute, University of California Los Angeles 607 Charles E. Young Drive East, Los Angeles, CA 90095, USA

[2] Department of Chemical and Biomolecular Engineering, University of California, Los Angeles 508 Portola Plaza, Los Angeles, CA 90095, USA

Small molecules and polymers characterization

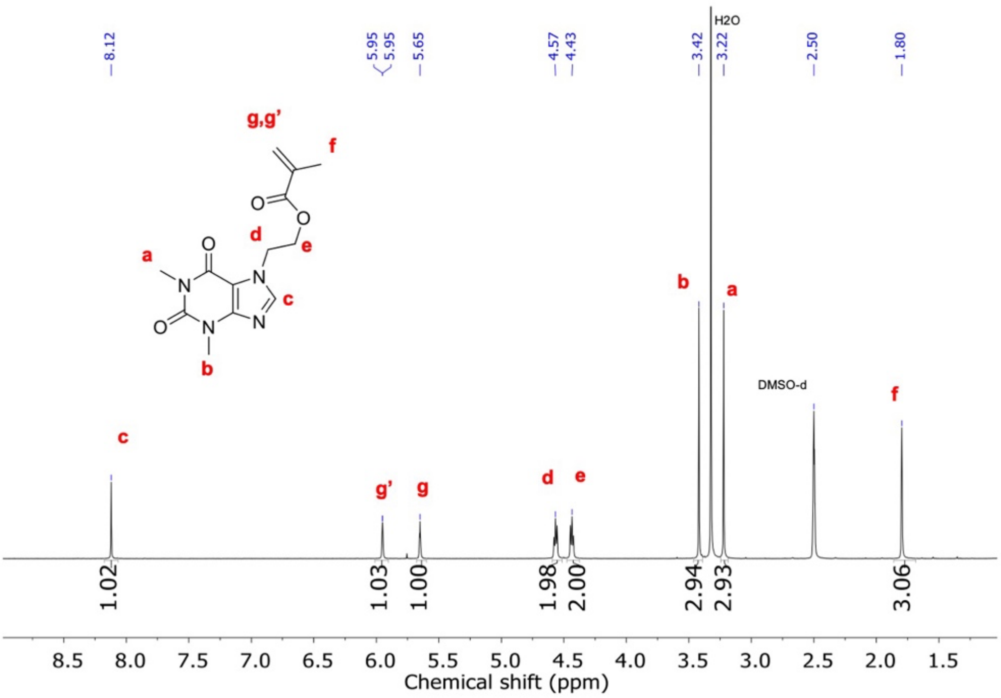

Figure S1. ^{1}H-NMR spectrum of **CafMA** in DMSO-d_6

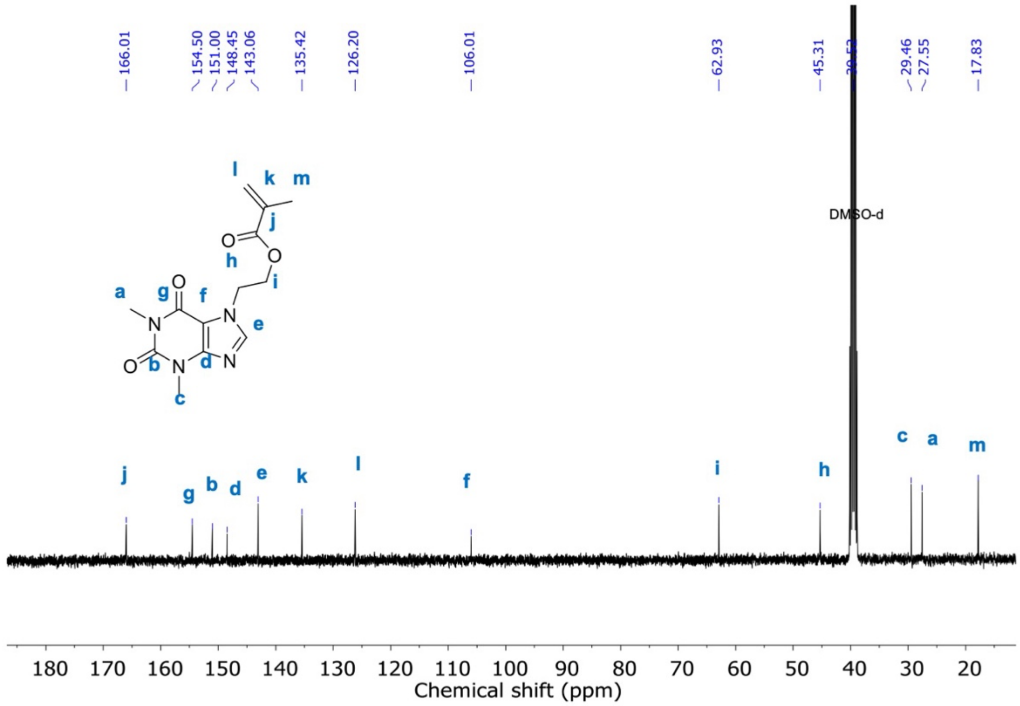

Figure S2. ^{13}C-NMR spectrum of **CafMA** in DMSO-d_6

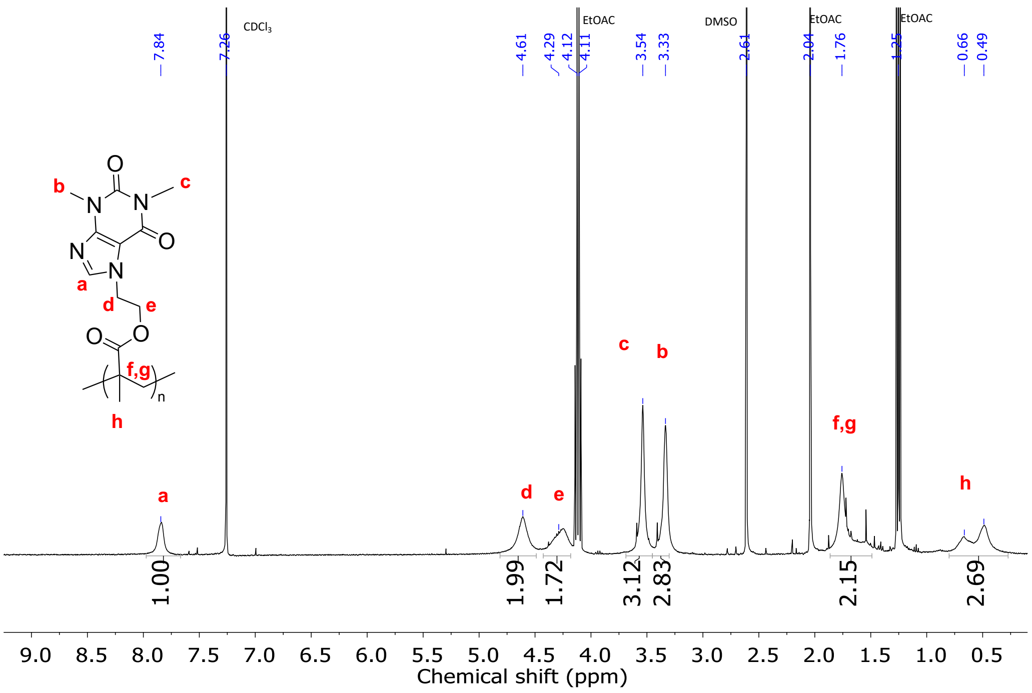

Figure S3. ^{1}H-NMR spectrum of **P(CafMA)** in $CDCl_3$.

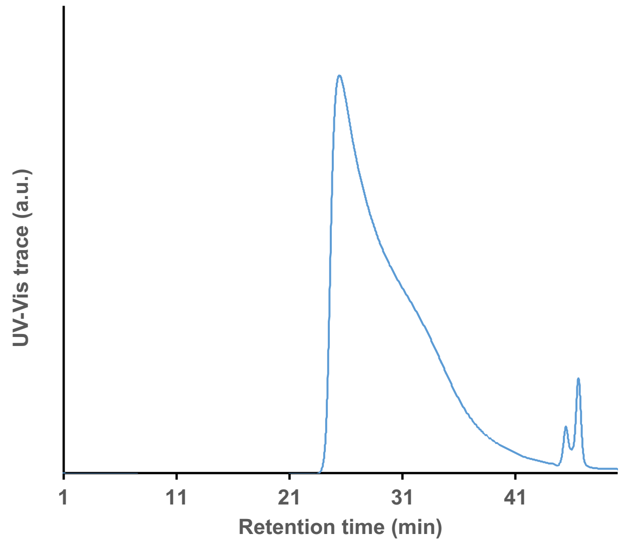

Figure S4. SEC chromatogram of **P(CafMA)** in trifluoroethanol.

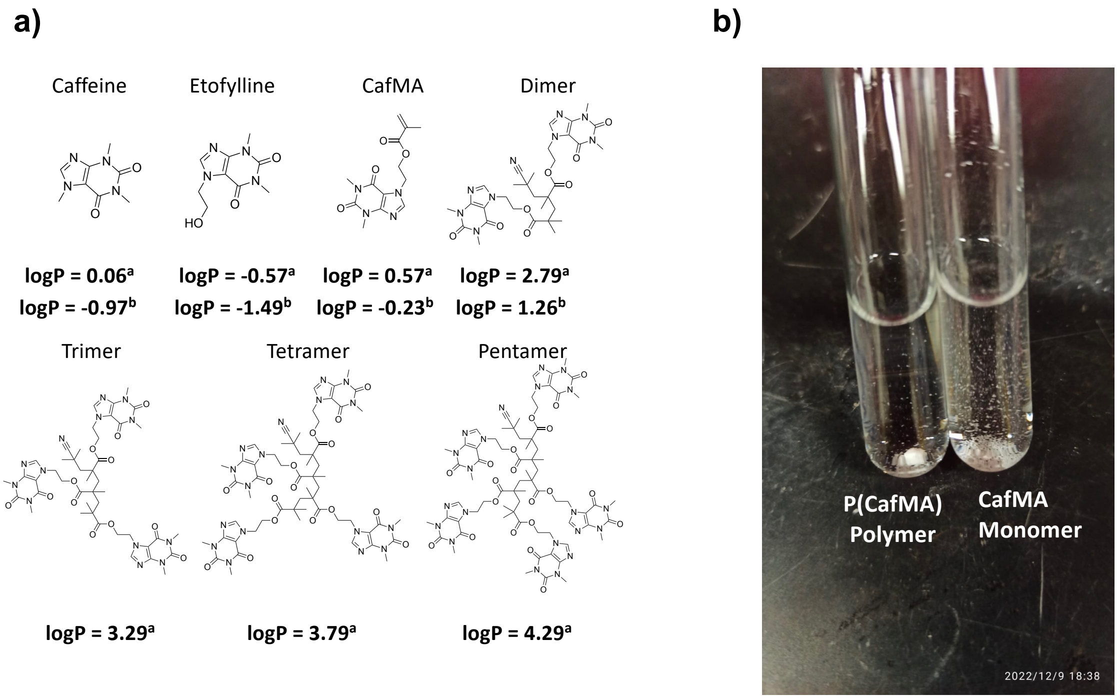

a. Using Chemdraw ®
b. Using molinspiration open source online

Figure S5. Solubility of monomer **CafMA**, oligomers and homopolymer **P(CafMA)**: a) theoretical study using ChemDraw® or molinspiration open source[1]; b) solubility in PBS at 1 mg/mL.

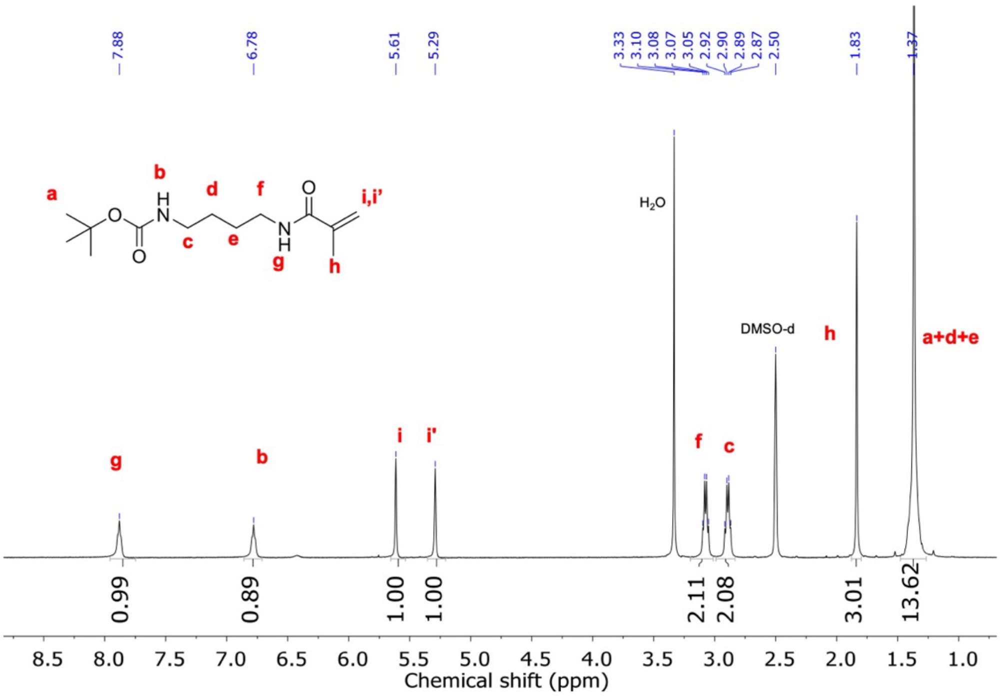

Figure S6. ^{1}H-NMR spectrum of **Boc-ab-MA** in DMSO-d_6

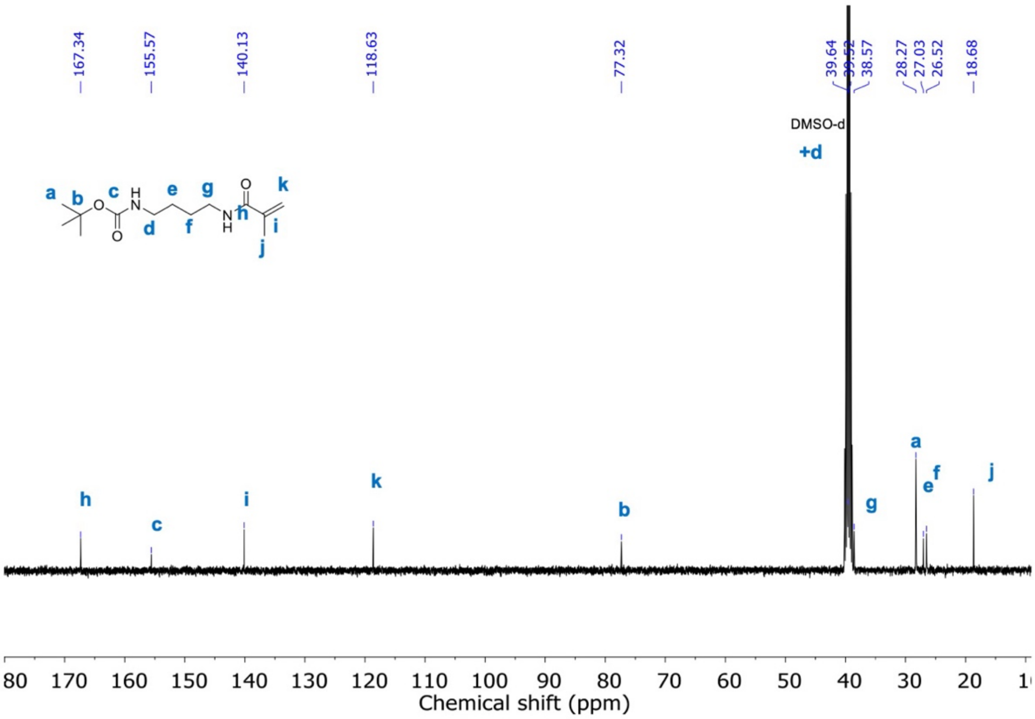

Figure S7. ^{13}C-NMR spectrum of **Boc-ab-MA** in DMSO-d_6

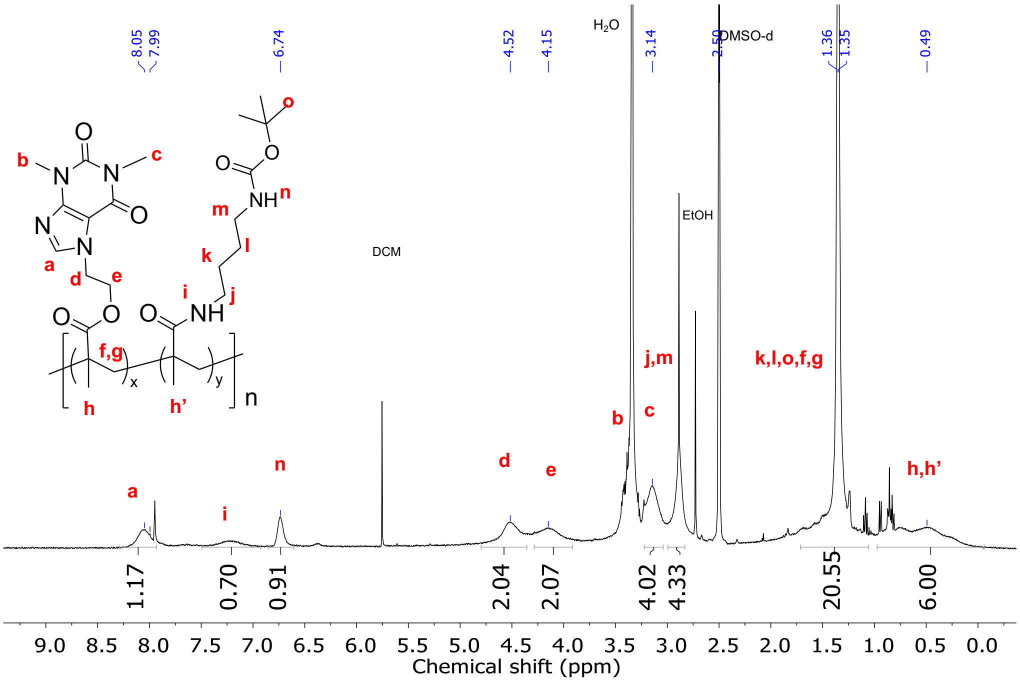

Figure S8. ^{1}H-NMR spectrum of **P(Boc-ab-Caf50%)MA** in DMSO-*d*$_6$

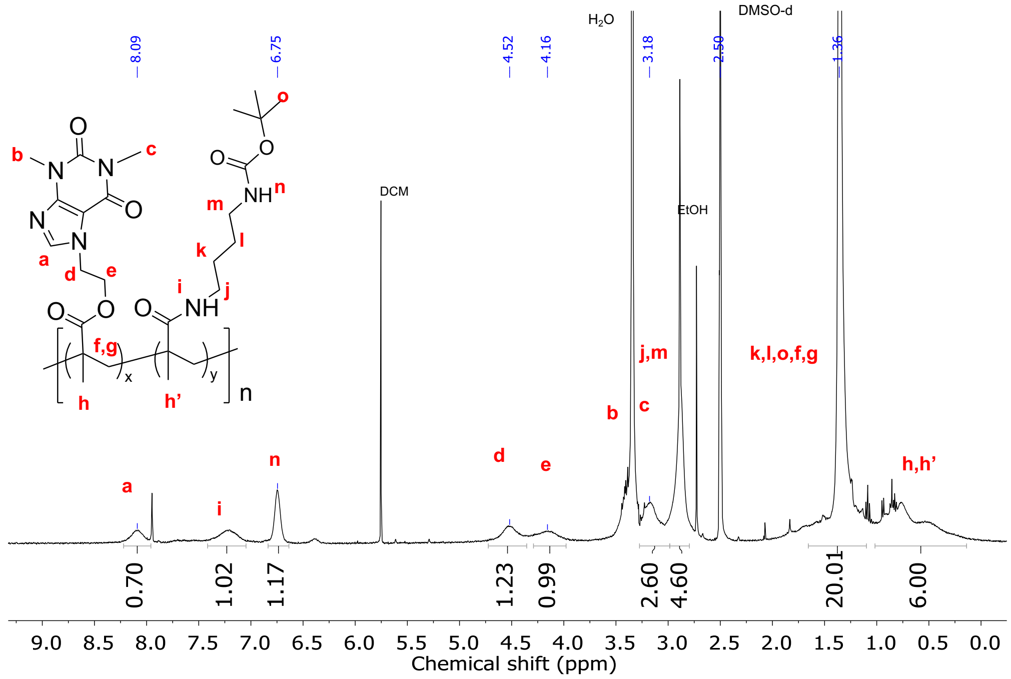

Figure S9. ^{1}H-NMR spectrum of **P(Boc-ab-Caf30%)MA** in DMSO-*d*$_6$

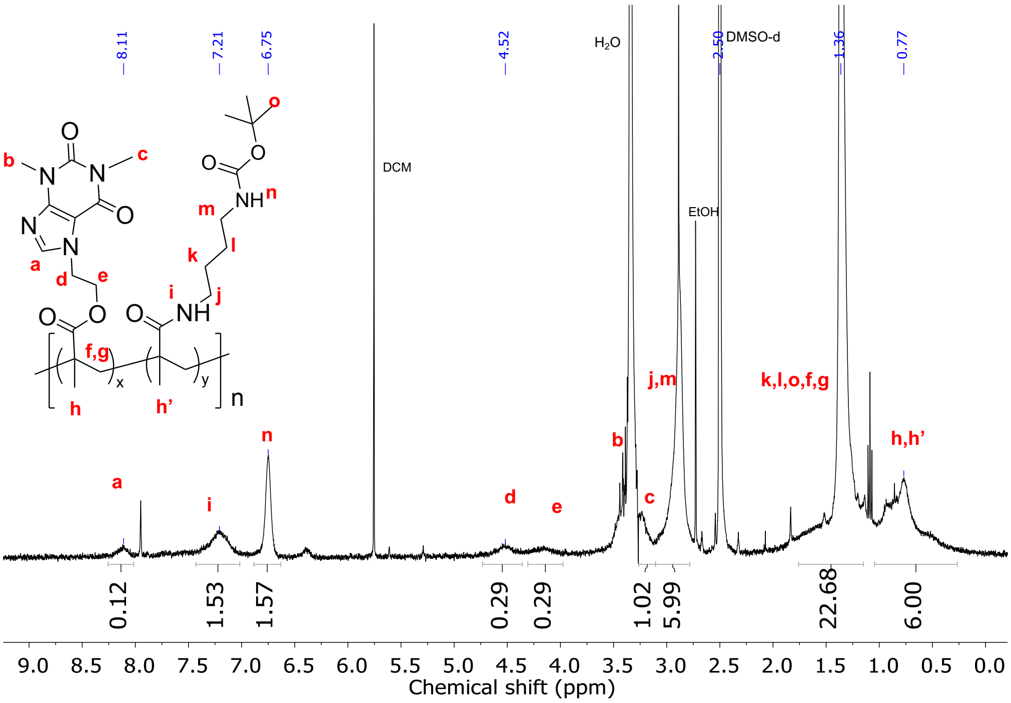

Figure S10. ^{1}H-NMR spectrum of **P(Boc-ab-Caf10%)MA** in DMSO-d_6

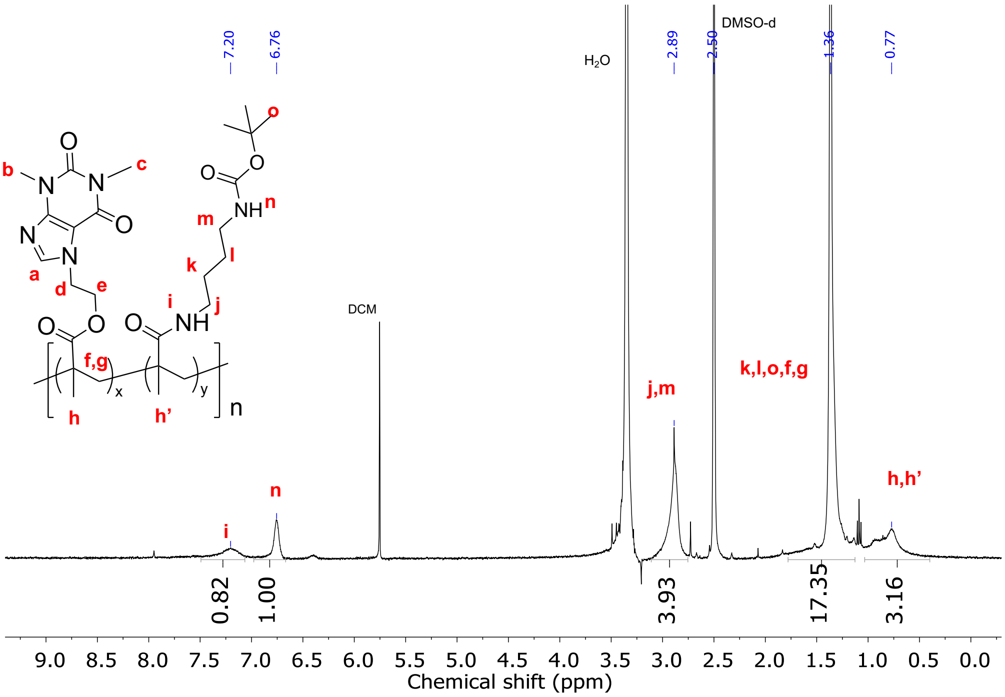

Figure S11. ^{1}H-NMR spectrum of **P(Boc-ab)MA** in DMSO-d_6

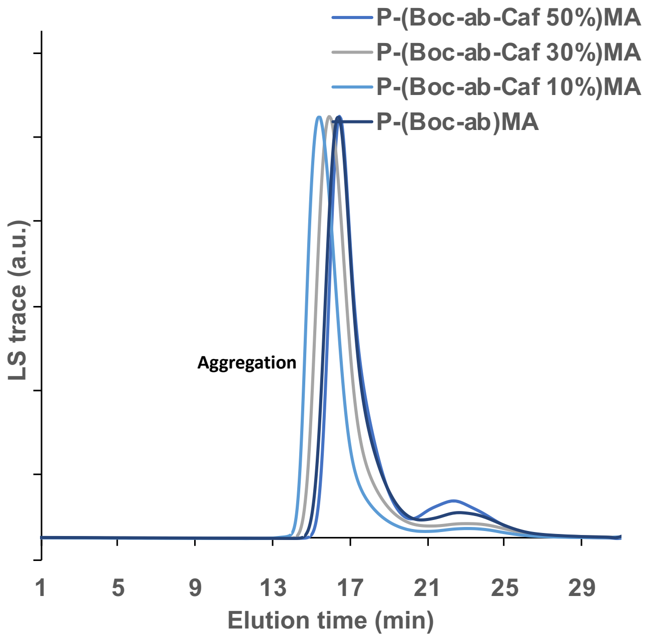

Figure S12. Light scattering signal (LS) from the series of copolymers **P(Boc-ab-Caf)MA** by SEC in DMF. The high signal from LS at lower elution time confirmed aggregation observed from the RI trace.

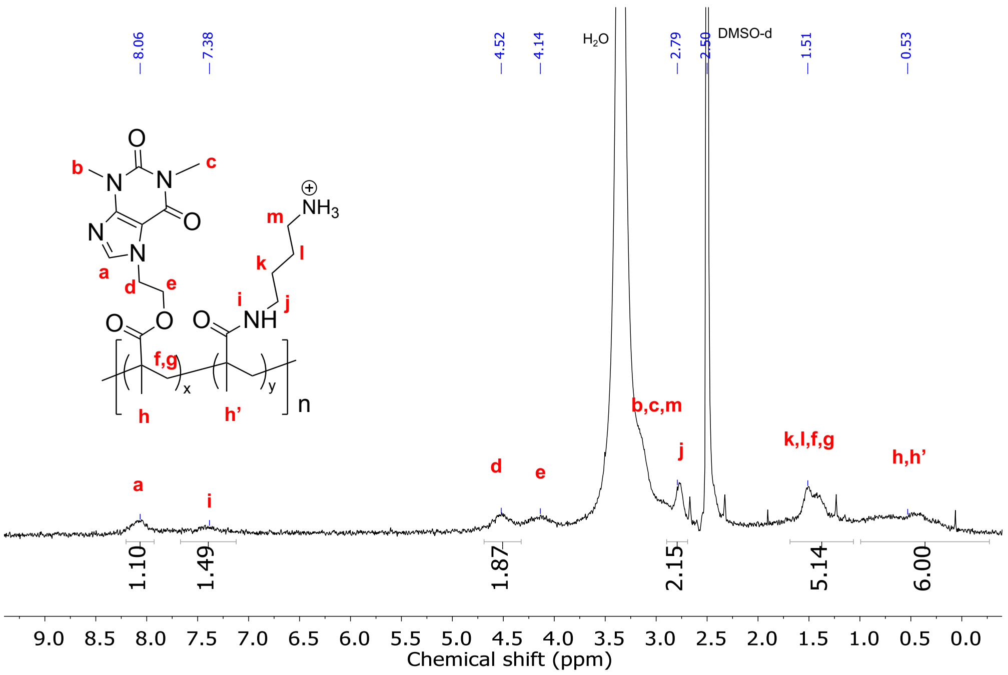

Figure S13. ^{1}H-NMR spectrum of **P(ab-Caf50%)MA** in DMSO-d_6

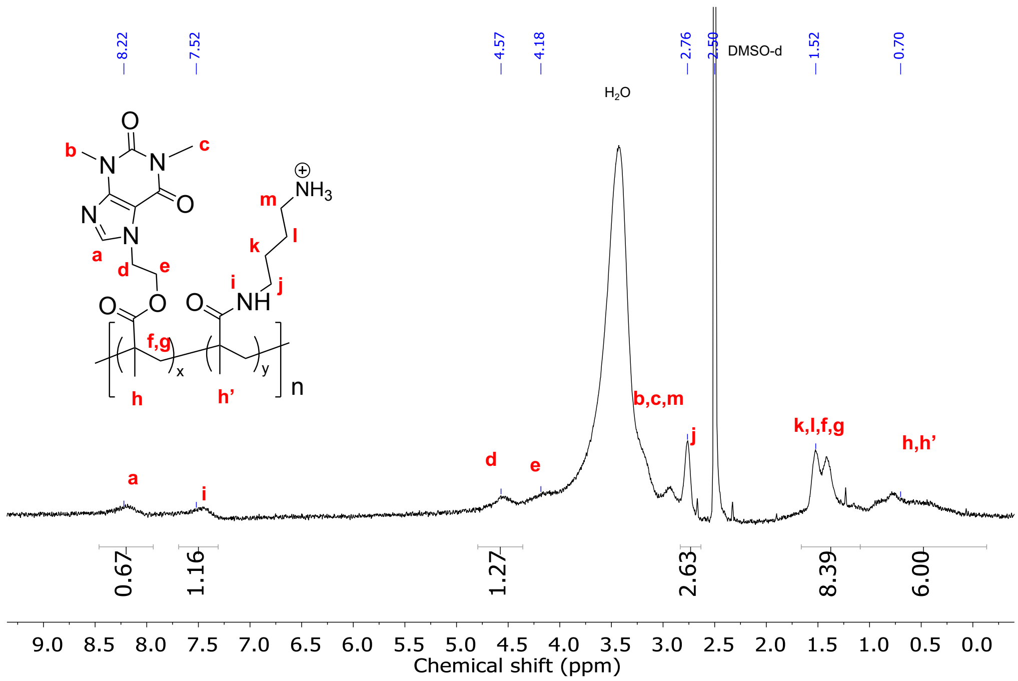

Figure S14. ^{1}H-NMR spectrum of **P(ab-Caf30%)MA** in DMSO-d_6

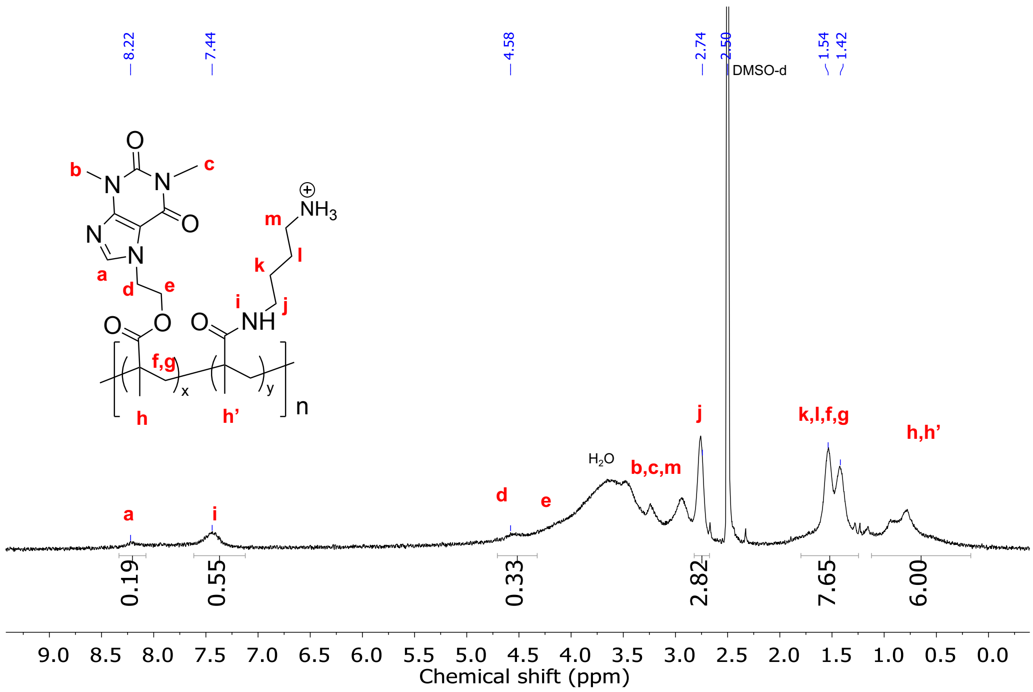

Figure S15. ^{1}H-NMR spectrum of **P(ab-Caf10%)MA** in DMSO-d_6

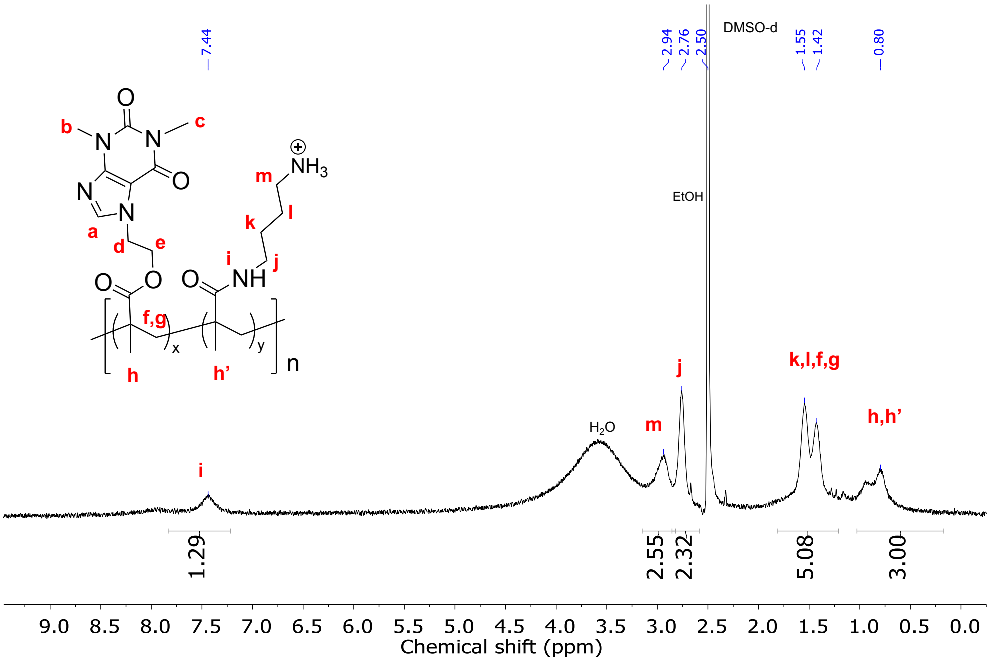

Figure S16. ^{1}H-NMR spectrum of **P(ab)MA** in DMSO-d_6

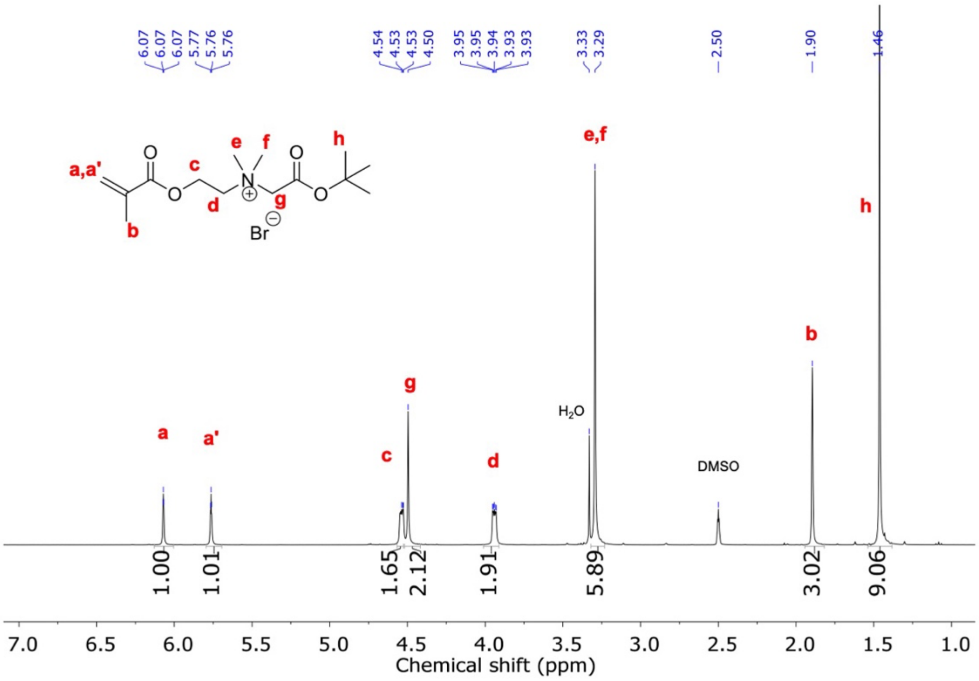

Figure S17. ^{1}H-NMR spectrum of ***t*-Bu-DMEA-MA** in DMSO-d_6

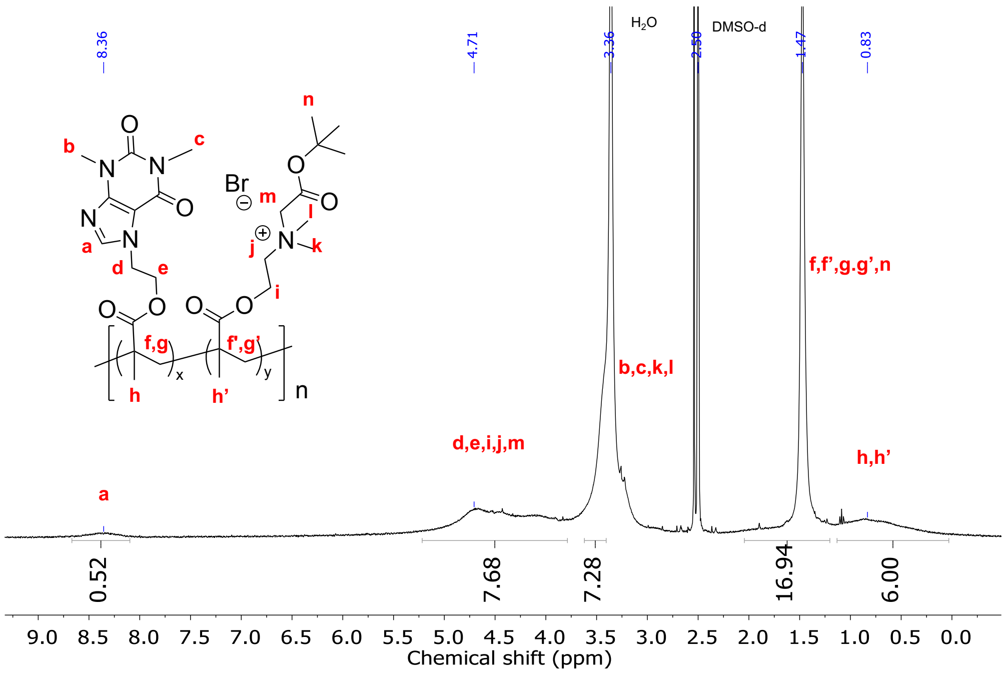

Figure S18. ^{1}H-NMR spectrum of **P(*t*-Bu-DMEA-Caf30%)MA** in DMSO-*d₆*

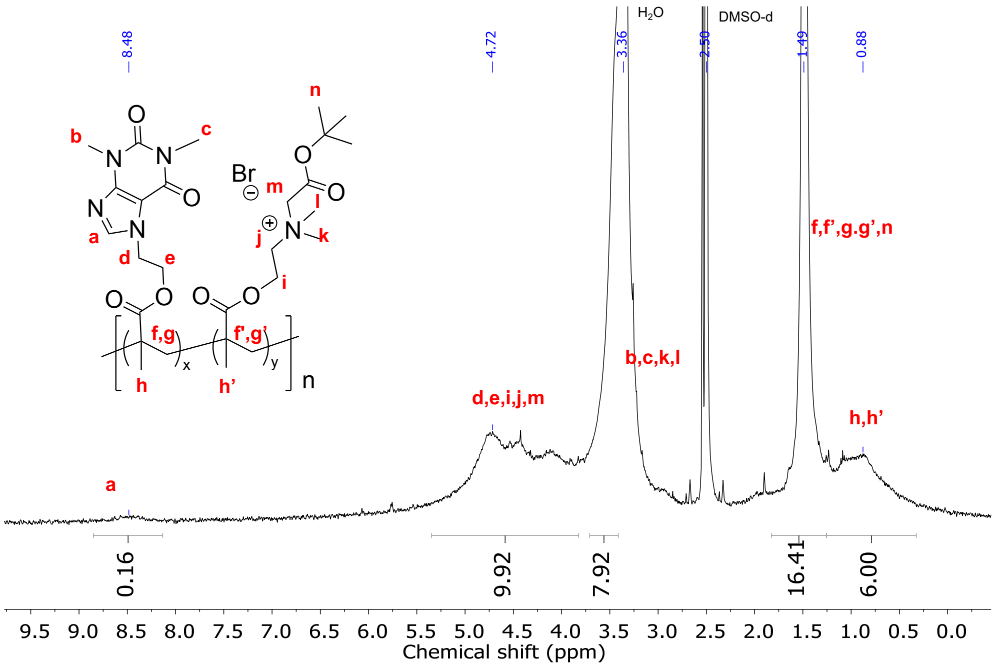

Figure S19. ^{1}H-NMR spectrum of **P(*t*-Bu-DMEA-Caf10%)MA** in DMSO-*d₆*

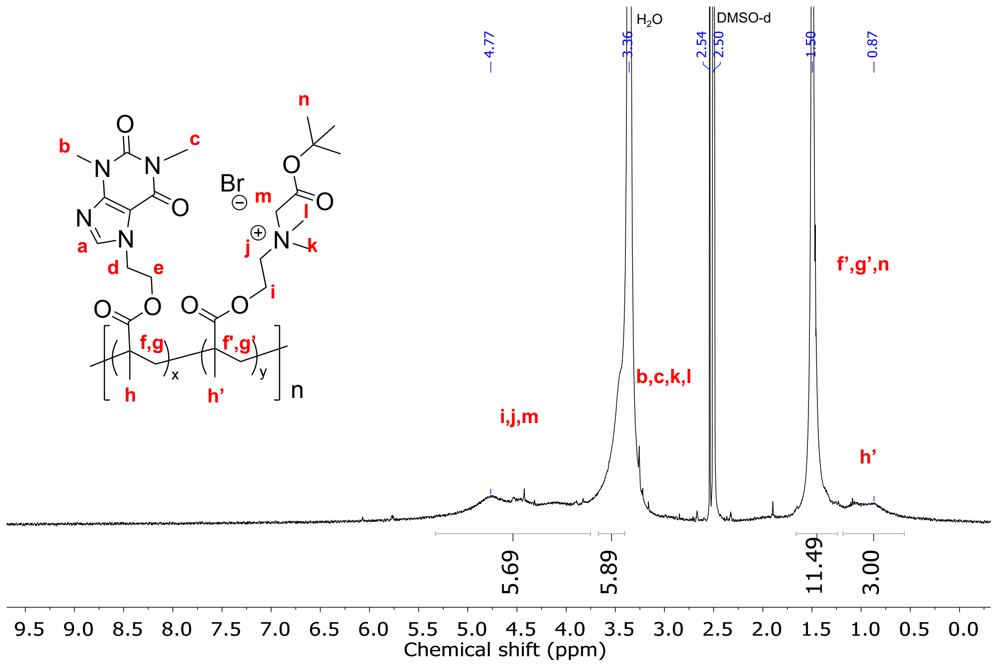

Figure S20. ^{1}H-NMR spectrum of **P(*t*-Bu-DMEA)MA** in DMSO-d_6

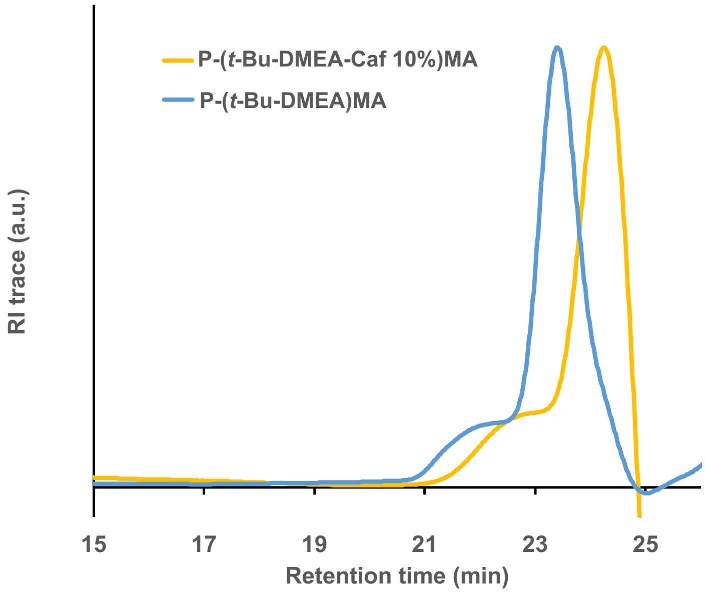

Figure S21. Chromatograms from aqueous SEC from RI detector for **P(*t*-Bu-DMEA-Caf)MA** series of copolymers.

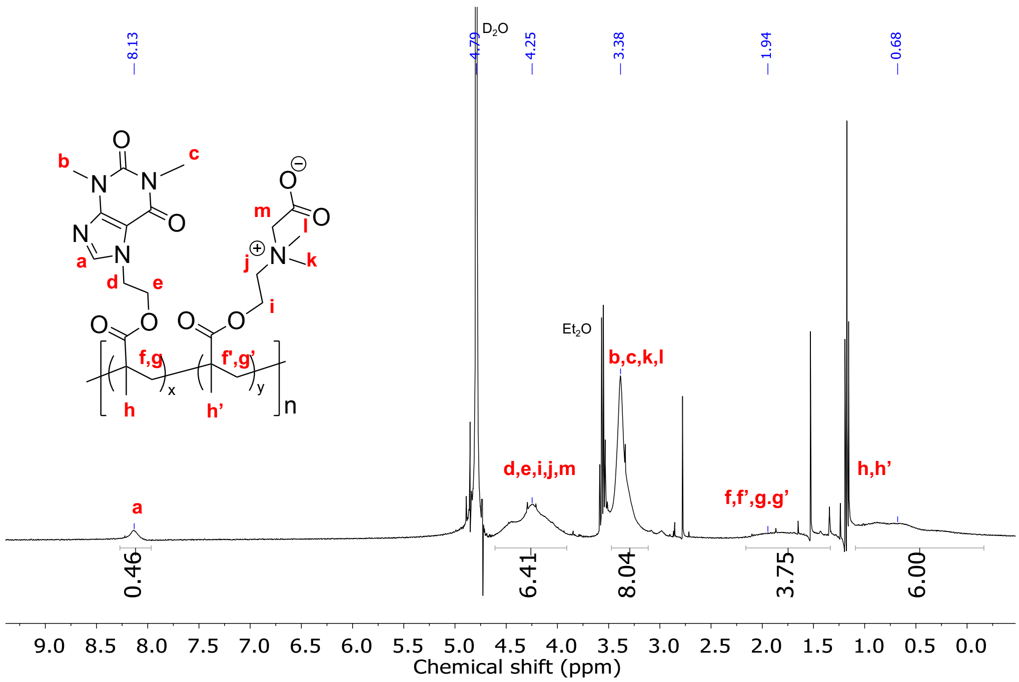

Figure S22. ^{1}H-NMR spectrum of **P(DMEA-Caf30%)MA** in DMSO-d_6

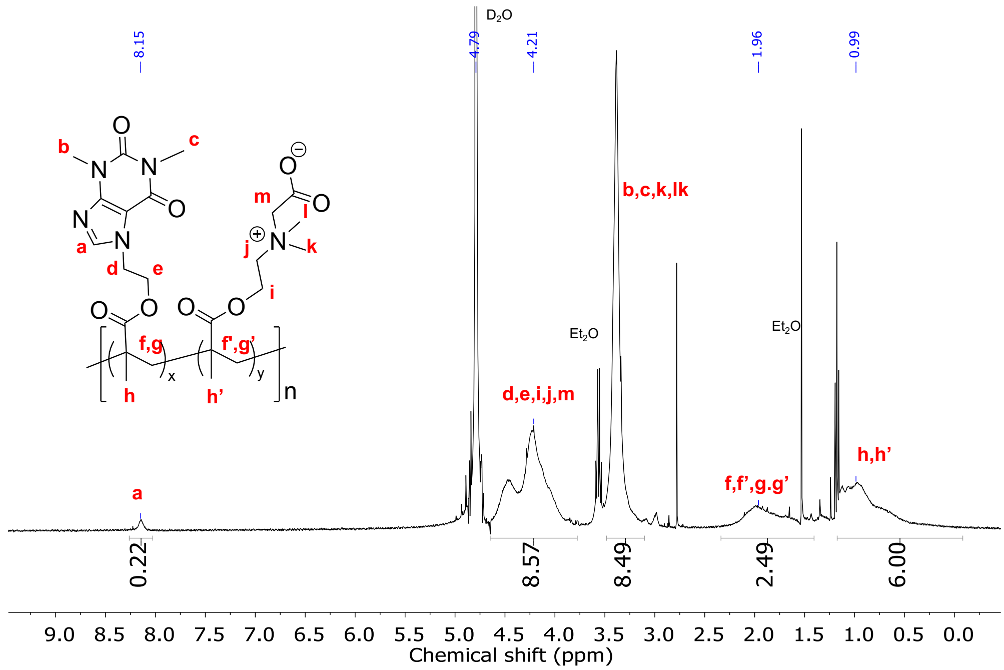

Figure S23. ^{1}H-NMR spectrum of **P(DMEA-Caf10%)MA** in DMSO-d_6

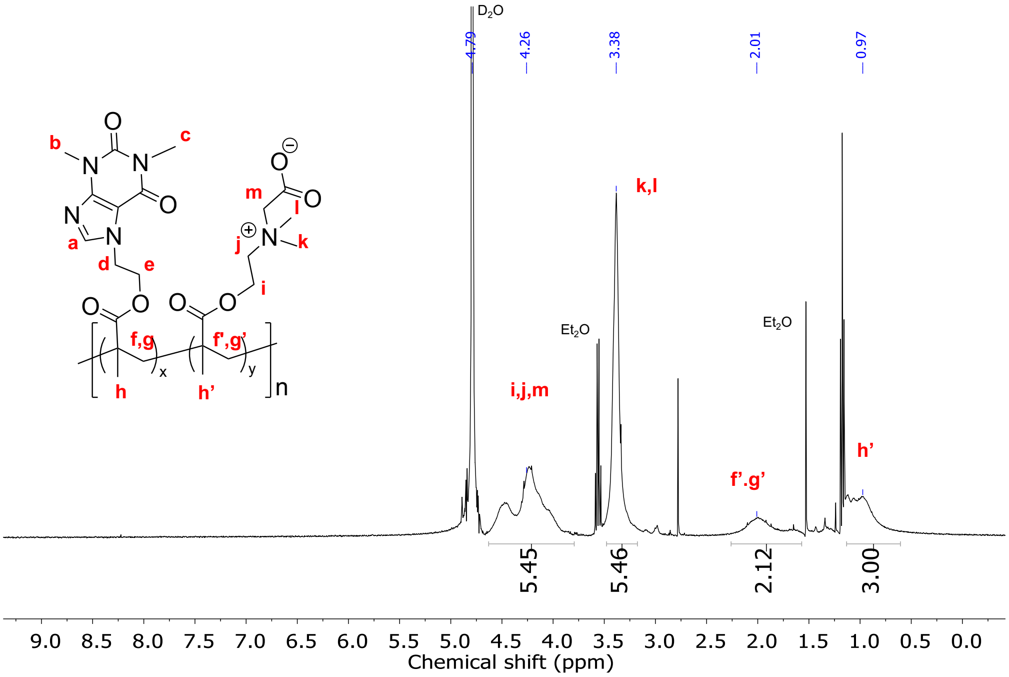

Figure S24. ¹H-NMR spectrum of **P(DMEA)MA** in DMSO-d_6

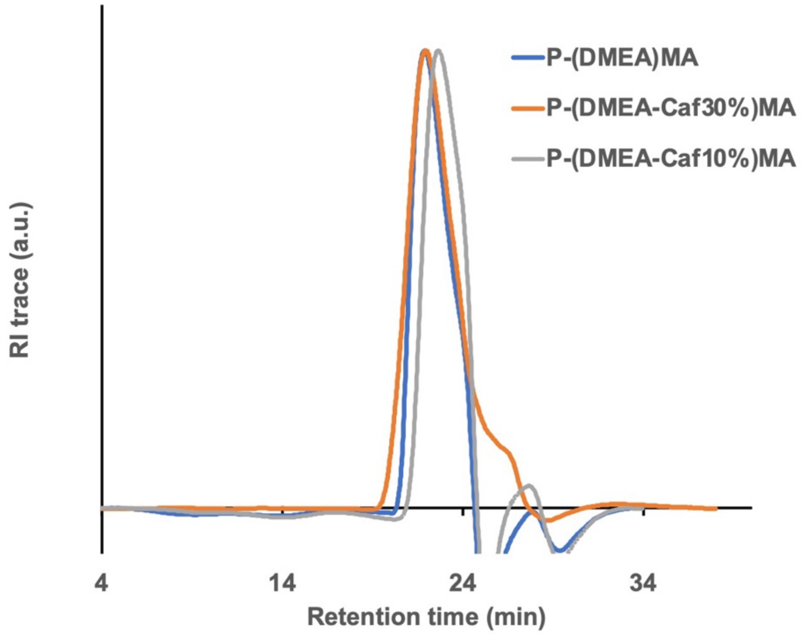

Figure S25. Chromatograms from aqueous SEC from RI detector for **P(DMEA-Caf)MA** series of copolymers upon Boc-deprotection.

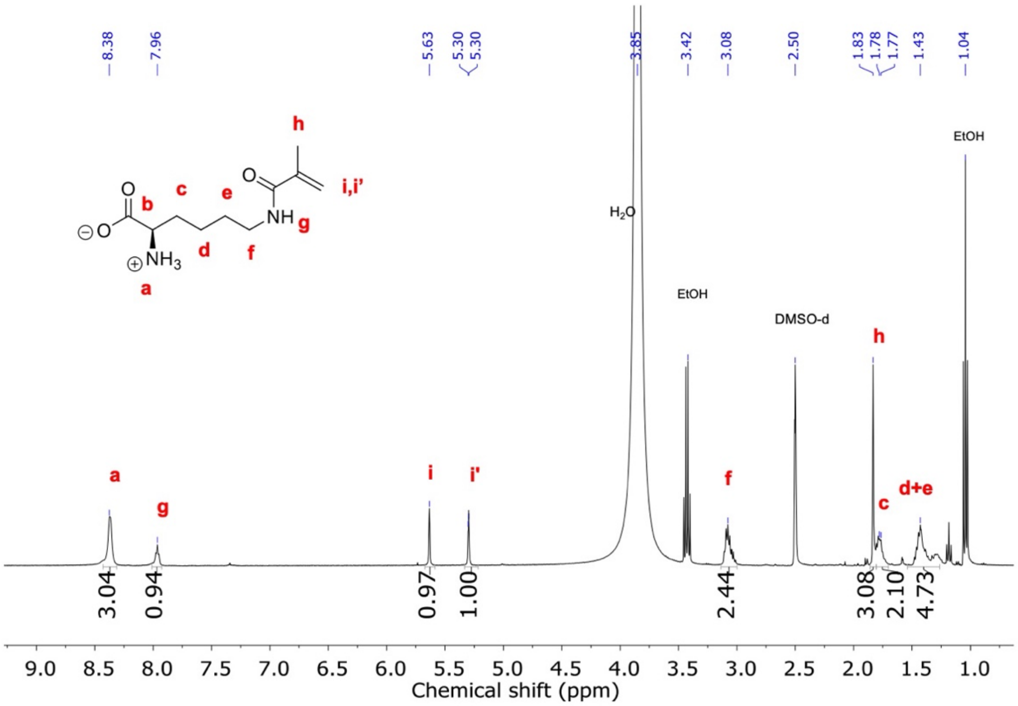

Figure S26. ^{1}H-NMR spectrum of **Lys-MA** in DMSO-d_6

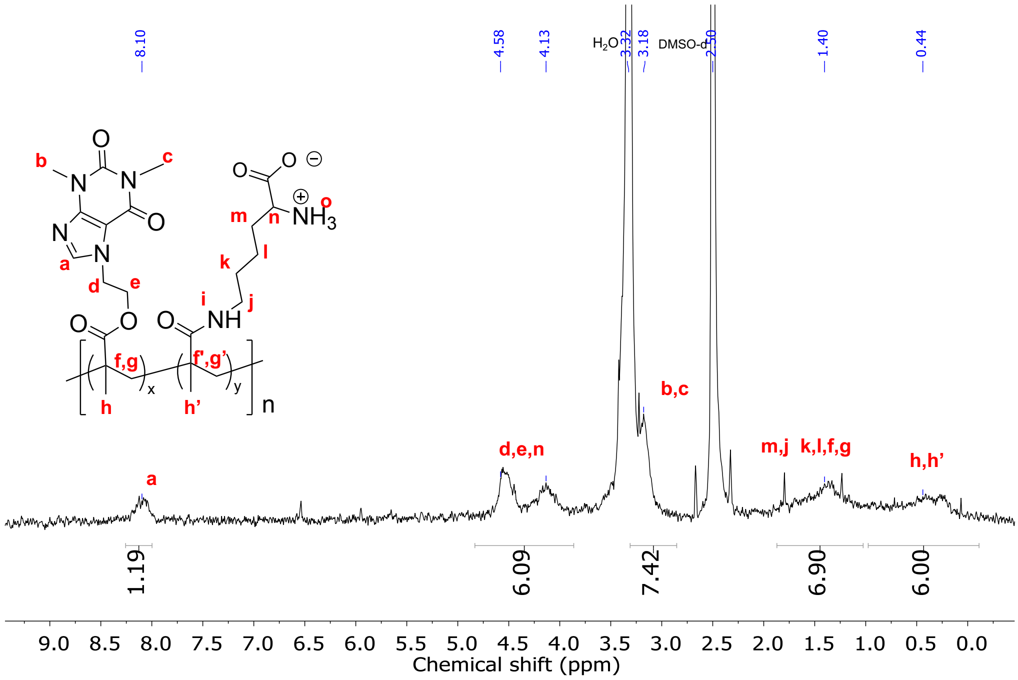

Figure S27. ^{1}H-NMR spectrum of **P(Lys-Caf50%)MA** in DMSO-d_6

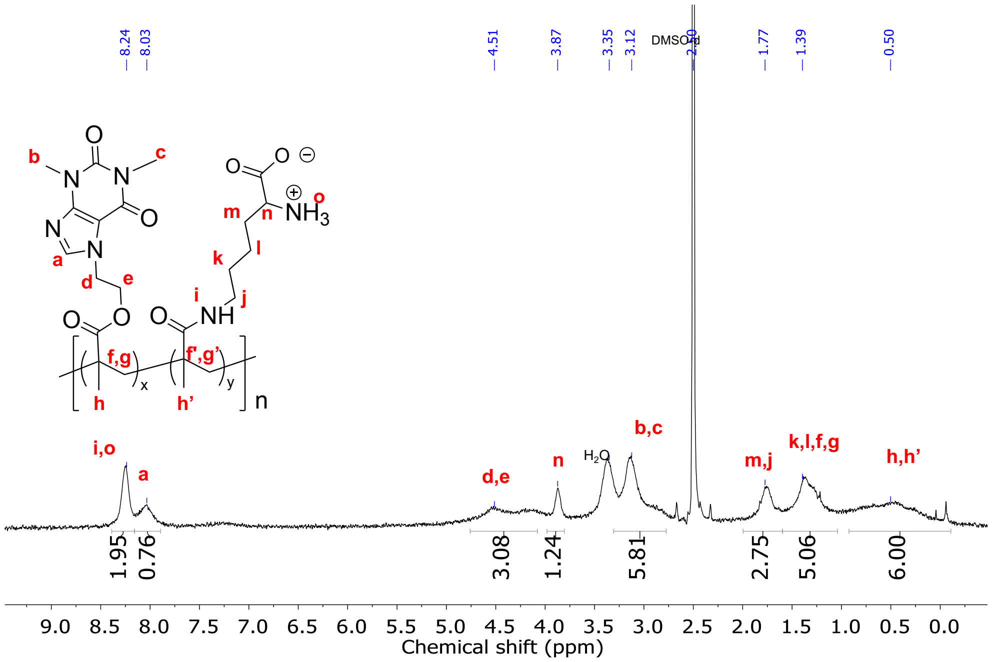

Figure S28. ^{1}H-NMR spectrum of **P(Lys-Caf30%)MA** in DMSO-d_6

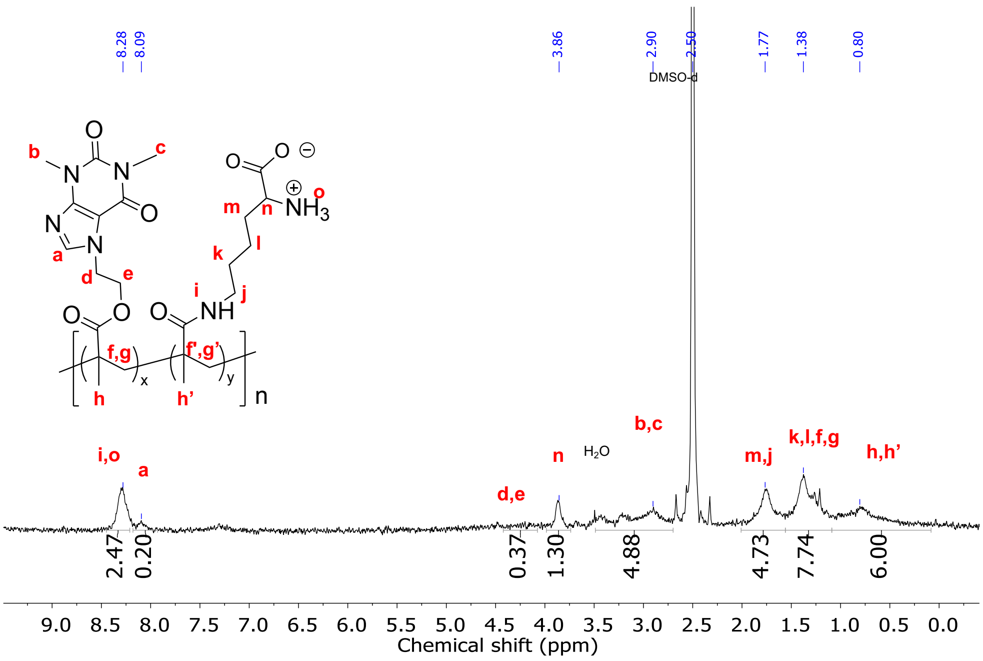

Figure S29. ^{1}H-NMR spectrum of **P(Lys-Caf10%)MA** in DMSO-d_6

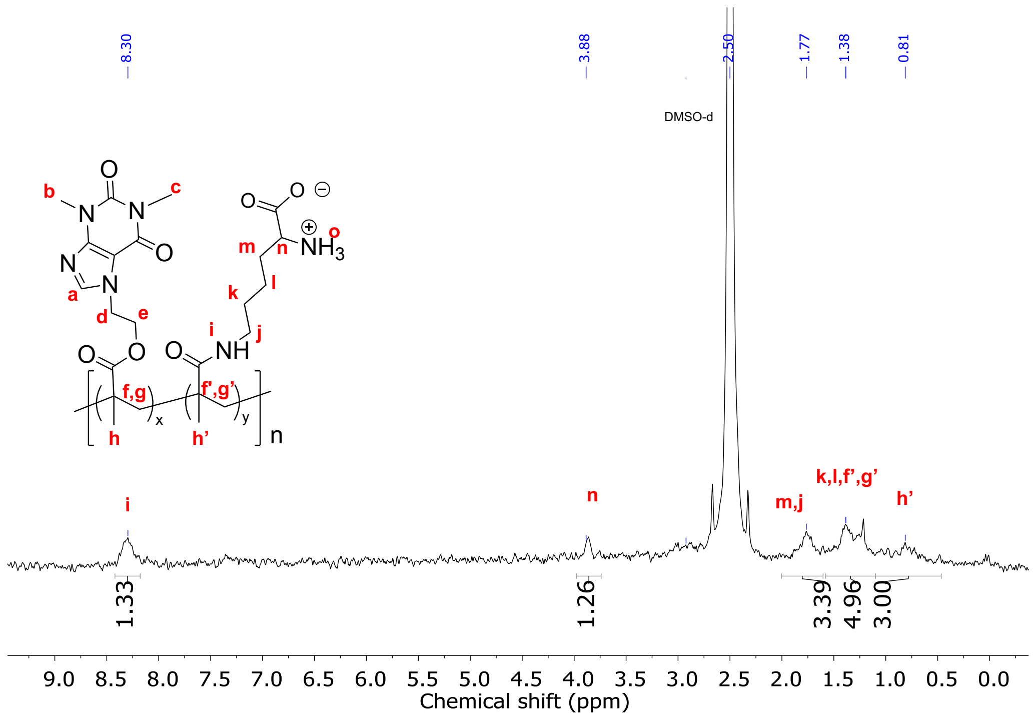

Figure S30. ^{1}H-NMR spectrum of **P(Lys)MA** in DMSO-d_6

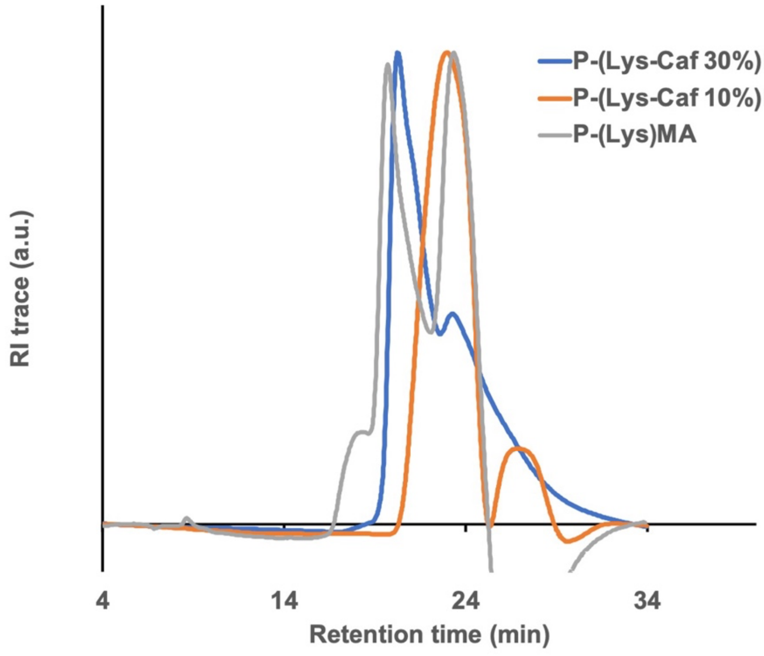

Figure S31. Chromatograms from aqueous SEC from RI detector for **P(Lys-Caf)MA** series of copolymers.

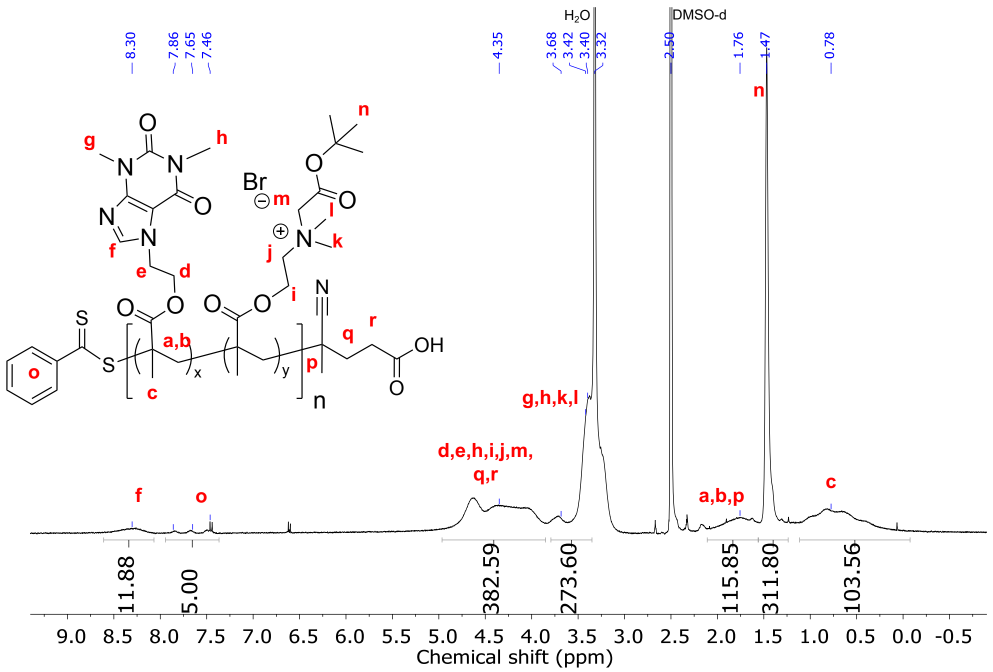

Figure S32. ^{1}H-NMR spectrum of **P(*t-Bu*-DMEA-Caf30%)MA DP30** in DMSO-d_6

DP= 87/3 = 29

% caff = 14%

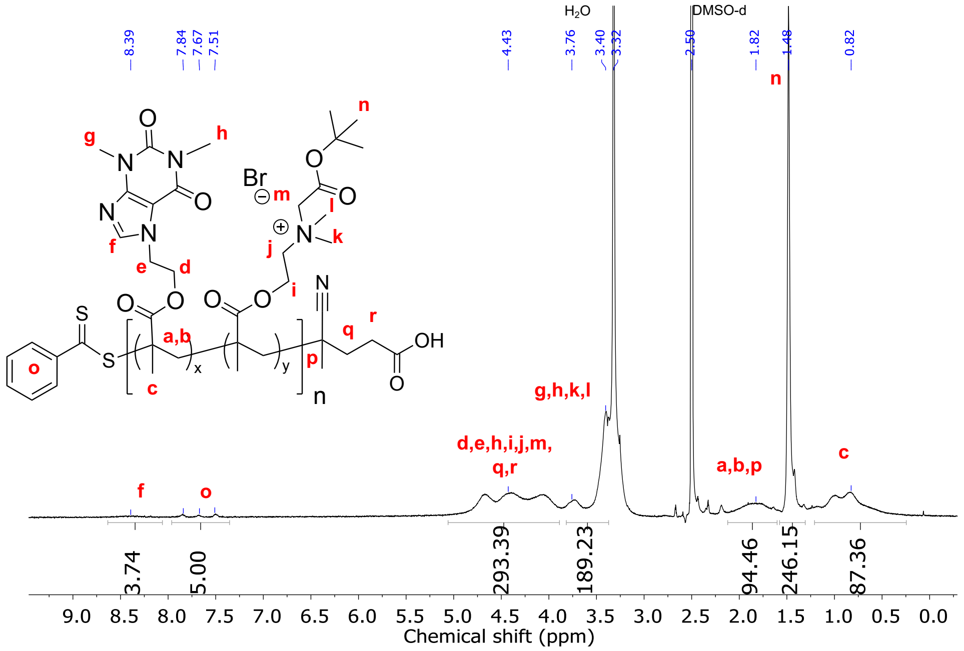

Figure S33. ^{1}H-NMR spectrum of **P(*t*-Bu-DMEA-Caf10%)MA DP30** in DMSO-d_6

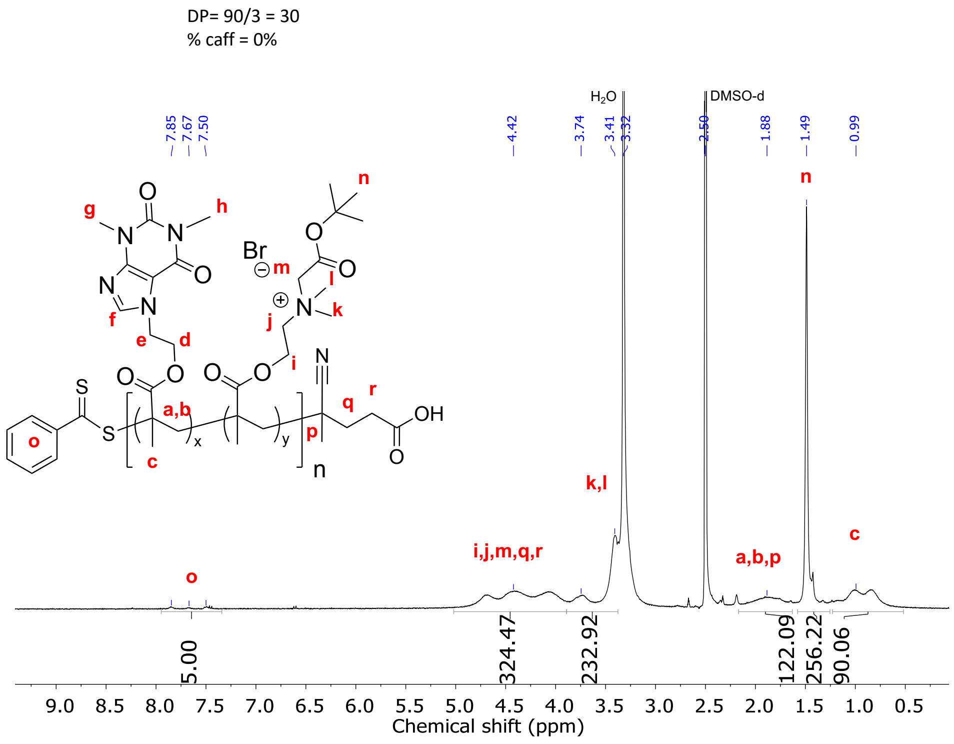

Figure S34. ^{1}H-NMR spectrum of **P(*t*-Bu-DMEA)MA DP30** in DMSO-d_6

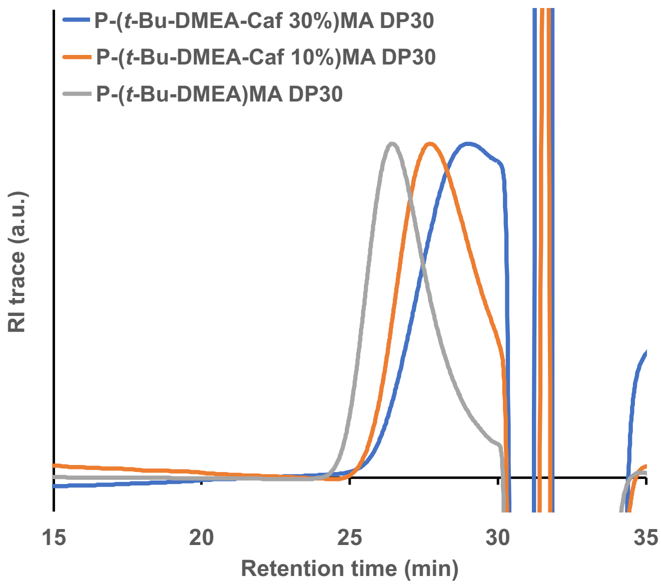

Figure S35. Chromatograms from aqueous SEC from RI detector for **P(*t*-Bu-DMEA-Caf)MA DP30** series of copolymers.

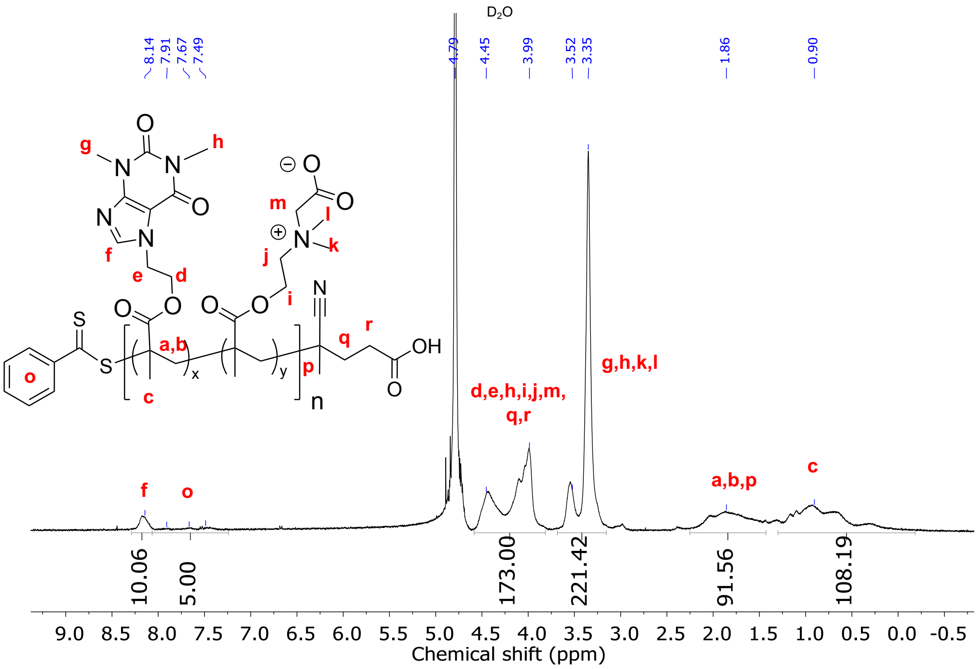

Figure S36. ^{1}H-NMR spectrum of **P(DMEA-Caf30%)MA DP30** in DMSO-d_6

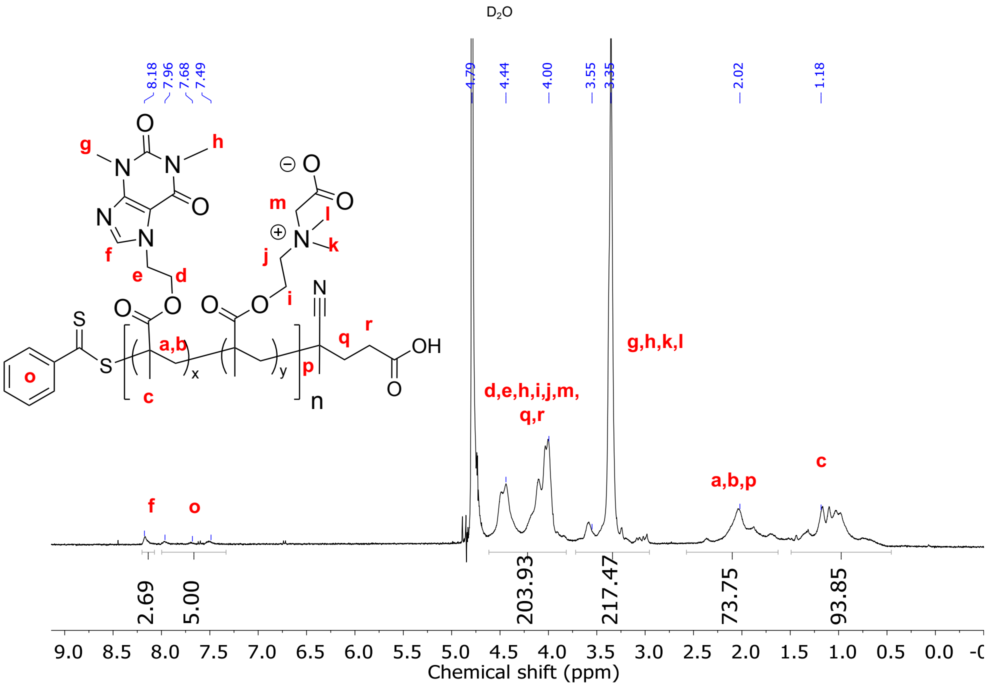

Figure S37. ^{1}H-NMR spectrum of **P(DMEA-Caf10%)MA DP30** in DMSO-d_6

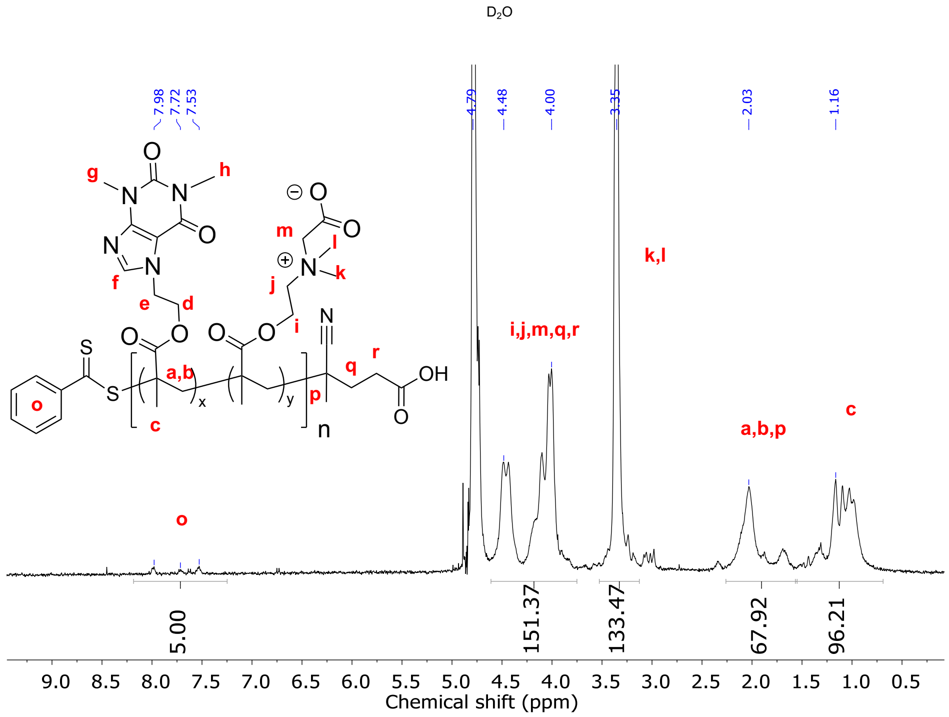

Figure S38. ^{1}H-NMR spectrum of **P(DMEA)MA DP30** in DMSO-d_6

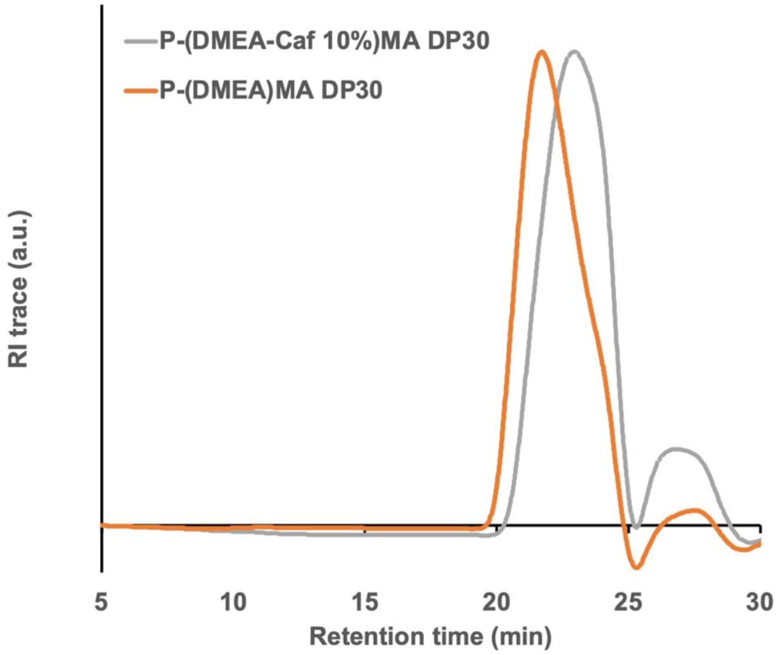

Figure S40. Chromatograms from aqueous SEC from RI detector for **P(DMEA-Caf)MA DP30** series of copolymers upon Boc-deprotection.

Biological results

Table S1. Antimicrobial evaluation of the caffeine copolymers against *S. aureus, E. coli and* methicillin-resistant *S. aureus* (MRSA) through the microdilution method by varying the caffeine content (Caf%) and the hydrophilic side chain. (n=6)

Polymer	*S. aureus* MIC (µg/mL)	*S. aureus* MIC (µM)[a]	*E. coli* MIC (µg/mL)	*E.coli* MIC (µM)[a]	MRSA MIC (µg/mL)	MIC MRSA (µM)[a]
**P-(ab-Caf 50%)MA**	>500	>18	125	4.5	-	-
**P-(ab-Caf 30%)MA**	>500	>18	26 ± 2	2 ± 0.1	-	-
**P-(ab-Caf 10%)MA**	>500	>18	19 ± 7	7 ± 0.1	-	-
**P-(ab)MA**	>500	>18	18 ± 7	7 ± 0.3	-	-
**P-(*t*-Bu-DMEA-Caf 30%)**	>500	>25	125	6.3	-	-
**P-(*t*-Bu-DMEA-Caf 10%)MA**	60 ± 39	2.9 ± 1.9	39 ± 11	1.9 ± 0.5	237 ± 83[b]<br>124 ± 94[c]	11.3 ± 4.0<br>5.9 ± 4.5
**P-(*t*-Bu-DMEA)MA**	15 ± 7	0.5 ± 0.2	41 ± 13	1.3 ± 0.4	197 ± 108[b]<br>128 ± 97[c]	6.2 ± 3.4<br>4.0 ± 3.0
**P-(DMEA-Caf 30%)MA**	>500	-	>500	-	-	-
**P-(DMEA-Caf 10%)MA**	>500	-	>500	-	-	-
**P-(DMEA)MA**	>500	-	>500	-	-	-
**P-(Lys-Caf 50%)MA**	>500	-	>500	-	-	-
**P-(Lys-Caf 30%)MA**	>500	-	>500	-	-	-
**P-(Lys-Caf 10%)MA**	>500	-	>500	-	-	-
**P-(Lys)MA**	>500	-	>500	-	-	-
**P-(*t*-Bu-DMEA-Caf 30%)MA DP30**	>500	-	>500	-	-	-
**P-(*t*-Bu-DMEA-Caf 10%)MA DP30**	>500	-	>500	-	-	-
**P-(*t*-Bu-DMEA)MA DP30**	>500	-	>500	-	-	-
**P-(DMEA-Caf 30%)MA DP30**	>500	-	>500	-	-	-
**P-(DMEA-Caf 10%)MA DP30**	>500	-	>500	-	-	-
**P-(DMEA)MA DP30**	>500	-	>500	-	-	-
**Ampicillin**	1	2.9	15.6	44.6	625	1290
**Kanamycin**	2	4.1	2	4.1	19.5	56

[a] Calculated from the molecular weight obtained from SEC. [b and c] Two different MRSA strains isolated from patients.

Table S2. Characterization of the copolymers bearing caffeine and cationic or zwitterionic side chains and DP = 30 from RAFT polymerization by ^{1}H-NMR, SEC and yields.

**Polymer**	**DP theoretical**	**DP from NMR**	**Caffeine content theoretical (%)**	**Caffeine content from ^{1}H-NMR (%)**	**M_n (kg/mol)**	**Đ**	**Yield (%)**
**P-(*t*-Bu-DMEA-Caf 30%)MA DP30**	30	34	30	35	6.4	1.16	70
**P-(*t*-Bu-DMEA-Caf 10%)MA DP30**	30	29	10	14	6.3	1.06	71
**P-(*t*-Bu-DMEA)MA DP30**	30	30	0	0	4.7	1.03	76
**P-(DMEA-Caf 30%)MA DP30**	30	36	30	28	n.a.	n.a.	72
**P-(DMEA-Caf 10%)MA DP30**	30	31	10	9	4.2	1.16	73
**P-(DMEA)MA DP30**		32	0	0	5.1	1.18	74

n.a. = Not analyzed due to poor solubility under the analytical conditions.

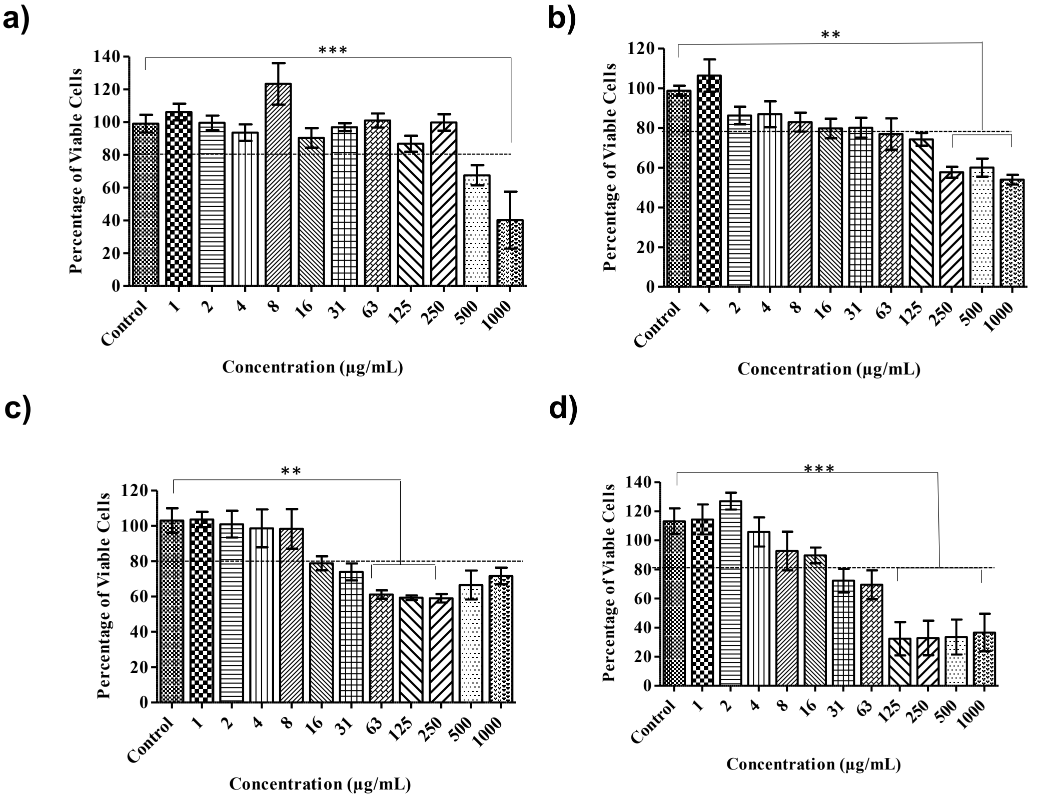

Figure S41. MTT Cell bioavailability results of a) **P-(ab-Caf50%)MA**, b) **P-(ab-Caf30%)MA,** and c) **P**) **P-(ab-Caf10%)MA** and d)) **P-(ab)MA** over NIH 3T3 fibroblast cells at 37 °C at 24 h. Data are represented as Mean ± SEM (n = 5). One-way ANOVA was performed followed by Tukey's test (***P < 0.001, **P < 0.01).

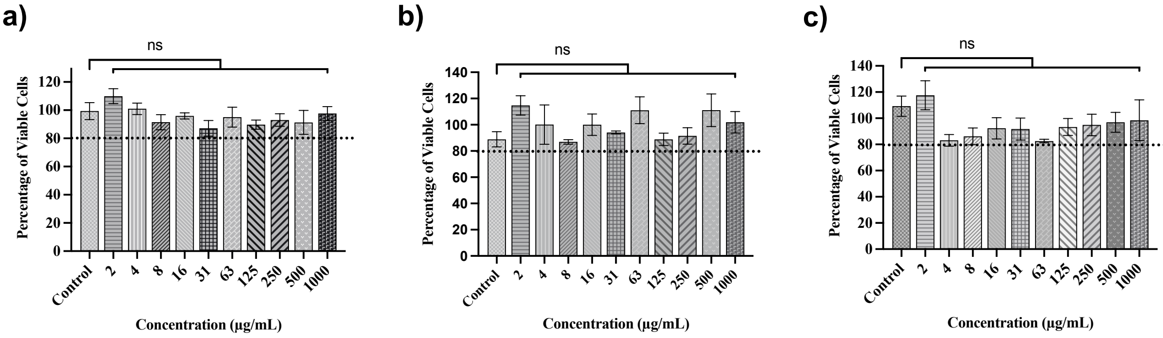

Figure S42. Cytotoxicity of a) **P-(DMEA-Caf30%)MA**, b) **P-(DMEA-Caf20%)MA,** and c) **P-(DMEA)MA** on NIH 3T3 fibroblast cell line after 24 h using MTT assay. Data are represented as Mean ± SEM (n = 3). One-way ANOVA was performed followed by Tukey's test.

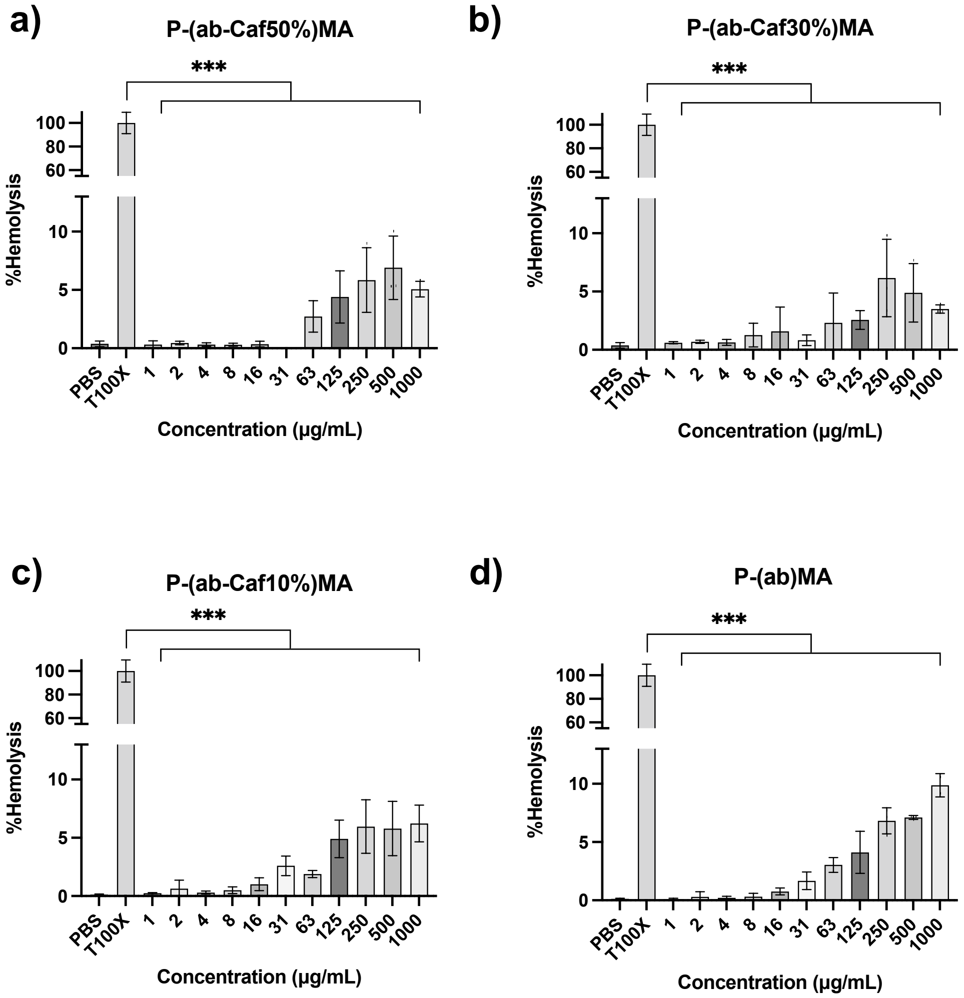

Figure S43. Hemolysis effect of a) **P-(ab-Caf50%)MA**, b) **P-(ab-Caf30%)MA,** and c) **P**) **P-(ab-Caf10%)MA** and d)) **P-(ab)MA**. Data are represented as Mean ± SEM (n = 3). One-way ANOVA was performed followed by Tukey's test (***P < 0.001).

References

(1) Molinspiration Property Calculation. Molinspiration Property Calculation https://www.molinspiration.com/services/faq.html (accessed Dec 21, 2022).
